# Effectiveness of Non-Pharmacological Interventions for Agitation during Post-Traumatic Amnesia following Traumatic Brain Injury: A Systematic Review

**DOI:** 10.1007/s11065-022-09544-5

**Published:** 2022-06-10

**Authors:** Sarah L. Carrier, Jennie Ponsford, Ruby K. Phyland, Amelia J. Hicks, Adam McKay

**Affiliations:** 1 Turner Institute for Brain and Mental Health, School of Psychological Sciences, Monash University, Melbourne, Australia; 2 Monash-Epworth Rehabilitation Research Centre, Epworth Healthcare, Melbourne, Australia; 3 Rehabilitation and Mental Health Division, Epworth Healthcare, Melbourne, Australia

**Keywords:** Agitation, Effectiveness, Inpatient care, Non-pharmacological intervention, Post-traumatic amnesia, Traumatic brain injury

## Abstract

Agitation is common in the early recovery period following traumatic brain injury (TBI), known as post-traumatic amnesia (PTA). Non-pharmacological interventions are frequently used to manage agitation, yet their efficacy is largely unknown. This systematic review aims to synthesize current evidence on the effectiveness of non-pharmacological interventions for agitation during PTA in adults with TBI. Key databases searched included MEDLINE Ovid SP interface, PubMed, CINAHL, Excerpta Medica Database, PsycINFO and CENTRAL, with additional online reviewing of key journals and clinical trial registries to identify published or unpublished studies up to May 2020. Eligible studies included participants aged 16 years and older, showing agitated behaviours during PTA. Any non-pharmacological interventions for reducing agitation were considered, with any comparator accepted. Eligible studies were critically appraised for methodological quality using Joanna Briggs Institute Critical Appraisal Instruments and findings were reported in narrative form. Twelve studies were included in the review: two randomized cross-over trials, three quasi-experimental studies, four cases series and three case reports. Non-pharmacological interventions were music therapy, behavioural strategies and environmental modifications, physical restraints and electroconvulsive therapy. Key methodological concerns included absence of a control group, a lack of formalised agitation measurement and inconsistent concomitant use of pharmacology. Interventions involving music therapy had the highest level of evidence, although study quality was generally low to moderate. Further research is needed to evaluate non-pharmacological interventions for reducing agitation during PTA after TBI.

**Systematic review registration number:** PROSPERO (CRD42020186802), registered May 2020.

**Supplementary Information:**

The online version contains supplementary material available at 10.1007/s11065-022-09544-5.

## Introduction

Agitated behaviours are frequently observed during the early recovery period following traumatic brain injury (TBI), known as ‘post-traumatic amnesia’ (PTA) or ‘post-traumatic confusional state’ (PTCS) (Bogner et al., 2001; Kadyan et al., 2004; Nott et al., 2006). A recent meta-analysis found that 44% of patients in PTA experience agitation, including restlessness, disinhibition, perseveration, impulsivity, emotional lability, confusion and verbal and physical aggression (Phyland et al., 2021). The disorientation and anterograde amnesia that is characteristic of PTA (Russell & Smith, 1961; Sherer et al., 2020; Stuss et al., 1999), is thought to impact patients’ ability to process and contextualise stimuli, resulting in inappropriate responses that manifest as agitation (Fugate et al., 1997; Harmsen et al., 2004; McKay et al., 2018; Noé et al., 2007).

Agitation is associated with poorer patient outcomes, including increased length of hospital stay, reduced engagement in rehabilitation, poorer cognitive and motor functioning and longer PTA duration (Bogner et al., 2001; Kadyan et al., 2004; Lequerica et al., 2007; Nott et al., 2006; Spiteri et al., 2021). Agitated behaviours increase the burden on staff and the risk of burnout; such behaviours can be disruptive and can pose a significant safety risk, and agitated patients are at increased risk of falls and often wander, thus requiring frequent supervision (Becker, 2012; Brooke et al., 1992; Montgomery et al., 1997; Sandel & Mysiw, 1996). Behavioural changes and longer PTA duration have also been associated with increased burden on family members, which can negatively impact the relationship between patients and families (Brooks et al., 1987; Norup et al., 2010). Agitation may cause distress for families, which can reduce their quality of life and ability to cope and provide adequate patient support (Norup et al., 2010). Given the significant impact of agitated behaviours on patients, their families and healthcare staff, the effective management of agitation during the PTA period is critical.

Evidence in support of effective intervention for managing agitation during PTA is lacking (Janzen et al., 2014; McNett et al., 2012; Mortimer & Berg, 2017). Pharmacological agents are frequently used, including anticonvulsants, antidepressants, beta-blockers, narcotics, benzodiazepines, neuroleptics and anti-parkinsonian medications (Duraski, 2011; Fleminger et al., 2006; Francisco et al., 2007; Fugate et al., 1997; Harmsen et al., 2004), however, evidence for their efficacy is weak (Bayley et al., 2019; Hicks et al., 2018; Janzen et al., 2014; McKay et al., 2021; Mehta et al., 2018; Nash et al., 2019; Williamson et al., 2019). Further, some medications commonly used to manage agitation (such as antipsychotics, anticonvulsants, sedatives) have been associated with impaired cognition, delayed recovery and a paradoxical increase in agitation (Bogner et al., 2015; Flanagan et al., 2009; Folweiler et al., 2017; Harmsen et al., 2004; Hicks et al., 2018; Hoffman et al., 2008; Kline et al., 2008; McNett et al., 2012; Phelps et al., 2015; Williamson et al., 2019; Zafonte, 1997). Current recommendations suggest limiting the use of pharmacological agents, except in the presence of severe agitation and aggression (Bayley et al., 2019; Ponsford et al., 2014).

Non-pharmacological interventions are recommended as the first-line approach for managing agitation (Eisenberg et al., 2009; McNett et al., 2012; Ponsford et al., 2014; Wiart et al., 2016). Examples of non-pharmacological interventions include environmental modifications (e.g., familiarising and orienting information), program modifications (e.g., adequate rest breaks, consistent staffing), behaviour modification techniques (e.g., identifying antecedents, positive reinforcement) and education for staff and family (Flanagan et al., 2009; Khan et al., 2015; Ponsford et al., 2014; Wiart et al., 2016). However, there is a lack of evidence in support of the efficacy of these strategies and our understanding of what constitutes best practice is limited (Fugate et al., 1997; McNett et al., 2012). Guidelines on the use of non-pharmacological interventions for agitation are primarily based on expert consensus due to a lack of empirical evidence (ABIKUS, 2007; ERABI, 2018; Ponsford et al., 2014; Wiart et al., 2016), and there are no systematic reviews evaluating the evidence for non-pharmacological management strategies to date.

### Review Objective

The objective of this review was to evaluate the effectiveness of non-pharmacological interventions for managing agitation during PTA in adults who have sustained a TBI. The specific review question was “What is the effectiveness of non-pharmacological interventions for managing agitation during PTA in adults (aged 16 years and older) who have sustained a TBI?”.

## Methods

This review was conducted and reported in accordance with the Preferred Reporting Items for Systematic Review and Meta-Analysis (PRISMA) guidelines (Page et al., 2021; Online Resource 1) and an a priori protocol (Carrier et al., 2020). The review has been registered in the international prospective register of systematic reviews (PROSPERO; CRD42020186802). There were three deviations from the protocol (Table 1).

**Table 1:** Deviation from the published methodology in the protocol for this systematic review and justification

**Change from protocol**	**Justification**
Relevant extract: “Studies were included where agitation was not the presenting symptom but was measured as an outcome variable.” Change: Studies were excluded if agitation was not measured as a primary outcome and the purpose of the intervention was not to reduce agitation.	The purpose of this study was to review non-pharmacological interventions for reducing agitation after TBI. Studies whereby reducing agitation was not a primary aim often involved interventions specific to the needs of a particular population and agitation was monitored to ensure the intervention did not increase agitation. Including these studies would not be consistent with the aims of the review, which is to present interventions that are developed with the primary intent of reducing agitation for most patients with a TBI.
Relevant extract: “The Grading of Recommendations, Assessment, Development and Evaluation (GRADE) approach for grading the certainty of evidence will be reported.” Change: The GRADE level of evidence will not be reported.	Due to the lack of studies included in this review, and the low quality and heterogeneity of the included studies with respect to interventions used and outcomes measured, GRADE levels of evidence were not reported.
Change: Studies were excluded if they did not report on a primary outcome (i.e., change in agitation levels or harms).	As the purpose of this study was to review non-pharmacological interventions for reducing agitation, it was not considered sufficient for studies to report on a secondary outcome, such as fatigue, without mention of any primary outcome (i.e., changes in agitation levels or harms resulting from use of an intervention).

### Data Sources and Searches

An initial limited search of CENTRAL and PubMED was undertaken to identify relevant articles. Text words contained in titles and abstracts of relevant articles and search strategies of relevant systematic reviews were used to develop a search strategy for PubMed, which was adapted for each information source. The search strategy was developed by an information specialist, using key words (linked with Boolean operators) and controlled vocabulary, and designed to locate published and unpublished studies (Online Resource 2). The search strategy was peer-reviewed against the Peer Review of Electronic Search Strategies (PRESS) checklist.

The databases searched were: MEDLINE OVID SP interface (1946–May 2020), PubMed excluding MEDLINE (1946–May 2020), Cumulative Index to Nursing and Allied Health (CINAHL; 1937–May 2020), Excerpta Medica Database (EMBASE) excluding MEDLINE OVID SP interface (1974–May 2020), PsycINFO (1806–May 2020) and CENTRAL (until May 2020). Four key journals were reviewed online: Brain Injury (1987–February 2021), Journal of Neurotrauma (1988–February 2021), Neuropsychology (1987–February 2021) and Journal of Head Trauma Rehabilitation (1986–February 2021). The clinical trial registries, International Clinical Trials Registry Platform Search Portal and ClinicalTrials.gov were searched in May 2020 using the term ‘traumatic brain injury.’ Key authors were contacted to identify additional studies (n = 16). Reference lists, citations and related articles of all included studies were also screened for additional studies. All supplementary searching was undertaken by SC, who has completed training in systematic review methodology. The overall review was last assessed as up to date in February 2021.

### Inclusion Criteria

#### Types of Studies

This review considered experimental and quasi-experimental study designs including randomised controlled trials, non-randomised controlled trials, before-and-after studies and interrupted time-series studies. This review also considered analytical observational studies including prospective and retrospective cohort studies and case–control studies, case series, single-arm studies and case reports for inclusion. This review did not consider case series with only post-test outcomes, qualitative research, protocols, methodological papers, descriptive cross-sectional studies, mechanism-based reasoning studies, comparative studies without concurrent controls, cluster clinical trials where the unit of analysis is the cluster, epidemiological studies of incidence and prevalence and studies of treatment preferences. Only studies with a title and abstract published in English were included. Studies in which the full text was published in a language other than English were translated via the Cochrane Task Exchange network (see Acknowledgements). Studies were included irrespective of publication year.

#### Types of Participants

This review considered studies involving participants aged 16 years and older, of any sex, who exhibit agitated behaviours during PTA after sustaining a TBI. Studies whereby at least 80% of the sample were 16 years and older were considered. Traumatic brain injury had to be confirmed according to established criteria including brain imaging findings, Glasgow Coma Scale (GCS) score and/or PTA status. Studies with ABI populations were included if TBI results were reported separately or at least 80% of the sample had sustained a TBI. Studies involving patients with all TBI severities (mild, moderate and severe) were accepted if patients were in PTA at study commencement (i.e., baseline). Patients’ PTA status was determined based on reference to relevant descriptors (e.g., disorientation, confusion and amnesia), any PTA assessment tools used, time post-injury and setting (Hicks et al., 2018). Retrospective and prospective measurements of PTA were accepted. Studies with patients in and out of PTA at baseline were included if more than 50% of the sample were in PTA or if data could be disaggregated. Studies were included if the intervention targeted agitation broadly or behaviours reflective of agitation (e.g., restlessness, frustration, disinhibition, perseveration, impulsivity, emotional lability and aggression) (Amato et al., 2012; Lequerica et al., 2007; Sandel & Mysiw, 1996; Weir et al., 2006). Medical and nursing notes or logbooks were accepted if results were presented quantitatively; qualitative descriptions of behaviour change were not sufficient. Relevant settings were acute care and inpatient settings.

#### Types of Interventions

This review considered studies that evaluated non-pharmacological interventions for managing agitation, with no restriction on type, duration, frequency, timing of delivery or concurrent, uncontrolled interventions used. This included the clinical prescription of medications that were not part of the intervention. Studies reporting on mixed interventions (i.e., controlled use of pharmacological and non-pharmacological interventions) were included if data for the non-pharmacological intervention were reported separately.

#### Types of Comparators

This review considered studies that included all types of comparators, including control conditions, supportive or standard care, baseline phase and other non-pharmacological or pharmacological interventions.

#### Types of Outcomes

Primary outcomes of interest for this review were change in agitation severity during PTA (which could also be measured by change in restraints, pharmacology used, and amount of direct supervision/observation required), and harms resulting from non-pharmacological intervention. Secondary outcomes of interest were changes in arousal, cognitive functioning, mood and fatigue, length of stay, duration of PTA, functional outcomes, and family and staff burden. Studies were required to report on at least one primary outcome.

### Study Selection

Two independent reviewers (SC and RP) screened the titles and abstracts of all identified publications against the inclusion criteria. The full texts of selected citations were retrieved and assessed in detail against the inclusion criteria by the two reviewers. Disagreements that arose between the reviewers at each stage of the study selection process were resolved through discussion or with adjudication by a third reviewer (AM, JP).

### Data Extraction and Assessment of Methodological Quality

Data was extracted by the two reviewers using a customized data extraction tool based on the standardized tool from the JBI (Joanna Briggs Institute) System for the Unified Management, Assessment and Review of Information (JBI-SUMARI). The tool was piloted and refined early in the data extraction phase. Eligible studies were critically appraised by the two reviewers at the study level for methodological quality using standardized critical appraisal instruments from the JBI (Tufanaru et al., 2020). Authors of included studies were contacted to request additional information where needed (10 of the 15 authors contacted provided a response). All studies, regardless of their methodological quality, underwent data extraction and synthesis. Reviewers were not blinded to the journal titles, study authors or their institutions. A meta-analysis was not conducted due to heterogeneity of included studies in terms of the interventions used and outcomes reported.

## Results

### Literature Search

**Fig. 1:**
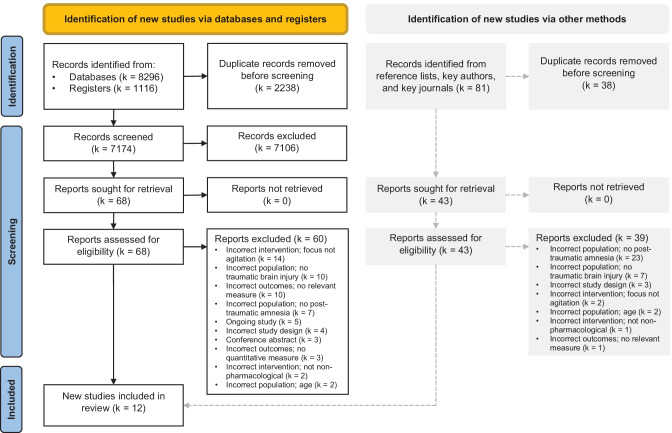
PRISMA flowchart detailing the results of the literature search, screening and study selection process

There were 7174 papers identified by the search strategy and 43 identified through other sources (i.e., reference lists of included studies, online review of key journals and via contact from study authors; Fig. 1). There were 7217 records in total after duplicates were removed. Of these, 7106 studies were excluded at the title and abstract screening stage. There were 111 studies screened at the full-text stage and 99 were excluded, leaving 12 studies eligible for inclusion (Fig. 1; Online Resources 3 and 4).

### Description of Included Studies

#### Study Design and Participant Characteristics

Twelve studies published between 1988 and 2019 were included in the review. There were two randomised cross-over trials which used a repeated measures cross-over design and the sample size ranged from 14 (Park et al., 2016) to 22 participants (Baker, 2001) (Table 2). There were three quasi-experimental studies (Formisano et al., 2001; Slifer et al., 1996, 1997); two used a multiple baseline design with sample sizes ranging from 3 to 6 and one used a single case design with a sample size of 34 (Table 3). There were four case series (Fluharty & Wallat, 1997; Magee et al., 2011; Nielsen et al., 2014; Wilson et al., 2019), with sample sizes ranging from 2 to 5 (Table 4) and three case reports (Berrol, 1988; Fluharty & Glassman, 2001; Kant et al., 1995) (Table 5). Participants’ ages ranged from 16 to 61 years. Traumatic brain injury severity varied across the included studies, although all participants had sustained at least a moderate to severe TBI (based on GCS and PTA duration). All participants were in PTA at the time of study commencement. Pharmacological interventions were frequently used alongside the non-pharmacological interventions under investigation. Studies were conducted in rehabilitation units, neurosurgical services, neuropsychiatric institutes, and intensive care units. Several studies included participants who did not meet inclusion criteria; data were extracted separately for relevant participants. Detailed critical appraisal results of included studies can be found in Online Resource 5.

**Table 2:** Study characteristics and findings from the randomized cross-over trials included in the review

**Study details**	**Participants**	**Intervention**	**Pharmacology**	**Outcomes**
Baker (2001) Private rehabilitation hospital, Australia Randomised crossover trial Risk of bias:^a^ Y: 6 N: 5 U: 2	**n:** 22 **Age:** M = 34 years, SD = 15.34 **Gender:** 17 males, 5 females **TBI severity:** Unclear, likely moderate to severe^b^ **Time post-injury:** Unclear **PTA:** WPTAS (< 9) **Agitation:** ABS	**Type:** Music intervention **Description:** Exposure to either live or taped music. Three music selections played at low volume **Setting:** Single bed hospital room **Frequency:** Maximum 15 min once per day **Duration:** 6 days (each condition repeated in random order)	Nil described	**Primary: Agitation (ABS)** Live music significantly lower than control condition (M = 5.01*, p* < .0001) Taped music significantly lower than control condition (M = 6.25, *p* < .0001) No significant difference between taped and live music (M = 1.20, *p* = .80) **Secondary: Orientation (WPTAS)** Live music significantly improved compared to control condition (estimated effect = 0.82, 95CI[0.39, 1.26], *p* < .001) Taped music significantly improved compared to control condition (estimated effect = 0.72, 95CI[0.29, 0.72], *p* < .001) No significant difference between taped and live music (estimated effect = -0.10, 95CI[-0.53, 0.33], *p* = .80)
Park et al. (2016) Brain injury rehabilitation unit, South Korea and USA Randomised crossover trial Risk of bias: Y: 9 N: 3 U: 1	**n:** 14 **Age:** M = 34.64 years, SD = 13.66, range = 19–61 **Gender:** 11 males, 3 females^c^ **TBI severity:** Severe (GCS < 8) **Time post-injury:** M = 40 days, SD = 23.15, range = 15–105 **PTA:** BNCE (< 27) **Agitation:** ABS	**Type:** Music intervention **Description:** Exposure to either preferred or classical relaxation music **Setting:** Bedside **Frequency:** Three hours per day (one hour no music, one hour music, one hour no music) **Duration:** 3 days (day 2 was a washout period)	Regular medication was indicated but not described. Data was not collected on days when a patient was given a non-regular, short-acting sedative medication	**Primary: Agitation (ABS)** Across time points Significant difference for preferred music (F = 5.53, *df* = 2, *p* = .01) Preferred music lower than baseline (*M* difference = 4.07, *p* = .02) Preferred music lower than post-intervention (*M* difference = -3.43, *p* = .03) No significant difference for classical relaxation music (F = 0.28, *df* = 2, *p* = .76) Between conditions Preferred music had significantly greater effect than classical relaxation music (*t* = -2.22, *df* = 12, *p* = .046) **Primary: Harms** Three patients exhibited increases in ABS scores during the preferred music condition

^a^*Y* low risk of bias, *N* high risk of bias, *U* unclear risk of bias, *N/A* item not applicable for study

^b^TBI severity was not reported but presumed to be at least moderate to severe based on PTA duration (all patients were in PTA during the intervention, which suggests PTA duration is greater than one day, thus excluding mild TBI)

^c^There is a discrepancy in the reporting of gender by Park et al. (2016). The text reports 11 males and 3 females, however, the table describes 12 males, 2 females

**Table 3:** Study characteristics and findings from the quasi-experimental studies included in the review

**Study details**	**Participants**	**Intervention**	**Pharmacology**	**Outcomes**
Formisano et al. (2001) Rehabilitation hospital, Italy Single case design Risk of bias: Y: 6 N: 2 N/A: 1	**n:** 7 included (n = 34 total)^a^ **Age:** M = 24.71 years, range = 15–42 **Gender:** 4 males, 3 females **TBI severity:** Severe (GCS < 8) **Time post-injury:** M = 254 days, range = 87–533 **PTA:** Unclear **Agitation:** Observations of video recordings using semi-quantitative scale of clinical modifications (‘improved,’ ‘unchanged’ or ‘worsened’)	**Type:** Music intervention **Description:** Exposure to improvised music therapy, through singing and use of instruments based on the patient’s pulse rate and breath **Setting:** Bedside **Frequency:** 20–40 min, three times per week **Duration:** Unclear, no longer than two months	No pharmacological intervention was changed during the intervention period. Treatments were not described	**Primary: Agitation** 7 out of 7 of the patients demonstrating psychomotor agitation had ‘improved’ following music therapy
Slifer et al. (1996) Paediatric neurorehabilitation unit, USA Non-concurrent multiple baseline design Risk of bias: Y: 6 N: 3	**n:** 1 included (n = 6 total)^b^ **Age:** 16 years **Gender:** Male **TBI severity:** Severe (GCS = 3) **Time post-injury:** 68 days **PTA:** GOAT (29 at intervention) **Agitation:** Percentage of therapies per day with one or more occurrences of ‘disruptive behaviour’, as determined by direct observation	**Type:** Behavioural strategies **Description:** Varied according to participants’ age, included positive reinforcement (immediate verbal praise, access to activities or tangible reinforcers at 15- and 30-min intervals), token economy, ignoring disruptive behaviours and response cost (loss of next scheduled activity or token) **Setting:** Ward **Frequency:** Delivered during 30-min therapy sessions, several sessions daily **Duration:** Approximately 21 days^c^	Nil described	**Primary: Agitation (% occurrence)** Reduced percentage occurrence of target behaviours during the intervention period (M = 22.3%) compared to the baseline period (M = 53%) **Secondary: PTA status (GOAT)** Baseline GOAT = 29, during differential reinforcement GOAT = 74, at discharge GOAT = 75
Slifer et al. (1997) Neurorehabilitation unit, USA Non-concurrent multiple baseline across subjects design Risk of bias: Y: 6 N: 3	**n:** 1 included (n = 3)^d^ **Age:** 16 **Gender:** Female **TBI severity:** Severe (GCS = 4) **Time post-injury:** 65 days **PTA:** Formal assessment (tool not described) **Agitation:** ABS, % scheduled therapy sessions attended, % intervals with disruptive behaviour	**Type:** Antecedent management and behavioural training delivered by 24-h behavioural assistant **Description:** Compliance training including close supervision, maintaining a quiet and calm environment and a behavioural protocol for disruption agitation and non-compliance. Patient was assigned a 24-h behavioural assistant **Setting:** Room / ward **Frequency:** Daily **Duration:** 72 days	Perphenazine (antipsychotic medication for first 32 days)	**Primary: Agitation (% of intervals with targeted disruptive behaviour)** Baseline: M = 42.3, SD = 35.2 CT + minimal demands: M = 12.1, SD = 10.6 CT + usual therapy: M = 7.3, SD = 10.3 **Primary: Agitation (ABS – daily mean ratings)**^e^ CT + minimal demands: M = 5.1, SD = 4.7 CT + usual therapy: M = 6.0, SD = 7.0 **Primary: Agitation (% of scheduled therapy sessions attended)** CT + minimal demands: M = 46.1, SD = 36.7 CT + usual therapy: M = 100, SD = 0

^a^Only the seven patients who presented as agitated and had sustained a TBI at commencement of the intervention were extracted from Formisano et al. (2001)

^b^Only TBI sample aged 16 years and older were extracted from Slifer et al. (1996)

^c^Duration of intervention estimated based on figure presented in Slifer et al. (1996)

^d^Pre-consultation baseline data was only available for 1 out of 3 participants, as behavioural psychology consultation was requested and initiated on the first day of scheduled therapies for the other two patients (i.e., there was no time to obtain pre-consultation baseline data in these cases). Only the participant with available baseline data was reported on

^e^The ABS in this study was scored according to the following criteria: 0 = absent, 1 = present to a slight degree, 2 = present to a moderate degree, 3 = present to a severe degree

**Table 4:** Study characteristics and findings from the case series included in the review

**Study details**	**Participants**	**Intervention**	**Pharmacology**	**Outcomes**
Fluharty and Wallat (1997) Rehabilitation institute, USA Case series Risk of bias: Y: 4 N: 5 N/A: 1	**n:** 1 included (n = 2)^a^ **Age:** 44 years **Gender:** Male **TBI severity:** Unclear, likely severe based on indicators of PTA at 7 months post-injury **Time post-injury:** 7 months **PTA:** Qualitative descriptors **Agitation:** Direct observation (average number of incidents of aggressive, agitated or uncooperative behaviour per day)	**Type:** Environmental modification **Description:** Staff addressed questions briefly, did not argue, staff walked away when patient ignored rules for conversation, encouraged to participate in tasks requiring full attention, embedded non-preferred tasks with enjoyable activities, locked ward **Setting:** Ward **Frequency:** Presumably every day **Duration:** 17 months	“High doses of haloperidol”	**Primary: Agitation (average number of incidents of aggressive, agitated or uncooperative behaviour each day)** At time of admission to NBP: 17 incidents per day (on average) At time of discharge (17 months post-admission): < 1 episode per day **Primary: Agitation (change in pharmacology)** At time of admission to NBP: “High doses of haloperidol” At time of discharge (17 months post-admission): no haloperidol, 20 mg of fluoxetine to blunt impulsive aggression
Magee et al. (2011) Hospital, Australia and UK Case series Risk of bias: Y: 2 N: 7 N/A: 1	**n:** 1 included (n = 6)^b^ **Age:** 26 years **Gender:** Male **TBI severity:** Severe (GCS = 5) **Time post-injury:** 5 weeks **PTA:** Formal assessment (measure not described) **Agitation:** Direct observation (number of sessions with decreased agitation and restlessness between 2-min pre- and post- observation periods)	**Type:** Music intervention **Description:** Exposure to live familiar music therapy, involving guitar and voice **Setting:** Bedside **Frequency:** 3 times per week (5–15 min) **Duration:** 4 weeks	Nil described	**Primary: Agitation (number of sessions with decreased agitation)** Degree of agitation and restlessness decreased between pre- and post- observation periods in 10 out of 12 sessions
Nielsen et al. (2014) Intensive care unit, Denmark Case series Risk of bias: Y: 6 N: 3 N/A: 1	**n:** 2 included (n = 5)^c^ **Age:** M = 43 years, range = 34–52 **Gender:** Male **TBI severity:** Unclear, likely severe **Time post-injury**: Case 4: 50 days, Case 5: 33 days **PTA:** CAM-ICU **Agitation:** Unclear, described agitation resolution (presumably using RASS and behavioural descriptions, including behaviours requiring restraint and high doses of antipsychotics)	**Type:** ECT **Description:** Bitemporal electrode placement with brief pulse square wave stimulation **Setting:** ICU **Frequency:** 3 days + 3 weekly maintenance treatments **Duration:** Case 4: 5 sessions total, case 5: 8 sessions total	Haloperidol, olanzapine, clonidine, midazolam, lorazepam	**Primary: Agitation resolution (RASS)** Case 4: Day 55 (5 days after ECT) Case 5: Day 40 (7 days after ECT) **Primary: Agitation (qualitative)** Case 4: Calm and cooperative, pharmacology discontinued Case 5: Delirious state terminated, cooperating, pharmacology discontinued **Primary: Harms** Case 4: In hospital at 6-month follow-up Case 5: Dead at 6-month follow-up (not related to ECT) **Secondary: PTA resolution** Case 4: Remained in PTA Case 5: 49 days
Wilson et al. (2019) Polytrauma rehabilitation centre, USA Case series Risk of bias: Y: 3 N: 6 N/A: 1	**n:** 2 **Age:** M = 27 years, range = 26–28 **Gender:** Male **TBI severity:** Severe (GCS < 9, LoC > 24 h) **Time post-injury:** Unclear, 14–23 days post-admission to rehabilitation centre **PTA:** O-Log **Agitation:** ABS and behavioural indicators (based on subjective report)	**Type:** Antecedent management and behavioural training **Description:** One session of CBT intervention involving ABC worksheets to identify and address maladaptive cognitions contributing to agitated behaviours (with assistance of psychologist) **Setting:** Ward **Frequency:** Once **Duration:** One occasion	Nil described	**Primary: Agitation** Case 1: Agitation and confusion remitted upon completion of ABC worksheet at day 14 (per medical chart, ABS and verbal report) Case 2: Much less agitated following worksheet (per parents, nursing team, and medical team report) **Secondary: PTA resolution** Case 1: Resolution of PTA one day after intervention (day 15) **Secondary: Sleep and anxiety** Case 2: Reduced hyperarousal and anxiety and improved sleep (no formal measurement used)

^a^Only the sample whose agitated behaviours were assessed in a quantitative manner were extracted from Fluharty & Wallat (1997)

^b^Only the TBI sample aged 16 years and older were extracted from Magee et al. (2011)

^c^Only the TBI sample was extracted from Nielsen et al. (2014)

**Table 5:** Study characteristics and findings from the case reports included in the review

**Study details**	**Participants**	**Intervention**	**Pharmacology**	**Outcomes**
Fluharty and Glassman (2001) Rehabilitation institute, USA Case report Risk of bias: Y: 7 N: 1	**n:** 1 **Age:** 23 years **Gender:** Male **TBI severity:** Unclear, likely severe **Time post-injury:** 18 months **PTA:** GOAT **Agitation:** Sum of verbally threatening and physically threatening behaviours and refusals of care across 20 5-day intervals, based on direct observation	**Type:** Antecedent management and behavioural training **Description:** Reframing agitating stimuli, making ADLs less noxious, one staff member for bathing, desensitisation to improve tolerance, use of Premack principles, encouraging therapeutic activities **Setting:** Ward and outdoors **Frequency:** Presumably every day **Duration:** Approximately 100 days (20 5-day intervals)	On admission: Tegretol 1400 mg qd., Inderal 80 mg tid., and Buspar 20 mg qid On discharge: Tegretol 500-mg bid. (to prevent seizures) and Ditropan 5-mg qhs. (to decrease bedwetting)	**Primary: Agitation (verbally threatening)** Sum of behaviours across 5-day intervals: 0, 2, 1, 0, 6, 3, 1, 0, 3, 1, 1, 1, 1, 0, 0, 2, 0, 0, 0, 2 **Primary: Agitation (physically threatening)** Sum of behaviours across 5-day intervals: 6, 1, 1, 0, 4, 5, 1, 0, 4, 0, 3, 1, 1, 0, 2, 1, 0, 0, 0, 1 **Primary: Agitation (refusal of cares)** Sum of behaviours across 5-day intervals: 4, 6, 3, 0, 1, 0, 2, 0, 0, 0, 3, 0, 0, 0, 0, 1, 0, 0, 0, 0
Berrol (1988) General hospital, USA Case report Risk of bias: Y: 4 N: 4	**n:** 1 **Age:** 19 years **Gender:** Male **TBI severity:** Unclear, likely severe **Time post-injury:** 26 days **PTA:** Qualitative descriptors **Agitation:** Nil, only harms described	**Type:** Physical restraint **Description:** Vest and soft restraints **Setting:** Bedside **Frequency:** Not described **Duration:** Approximately 8 days	Mildly sedated (medication not described)	**Primary: Harms** Found unconscious, dangling from vest and soft restraints without pulse or respiration. Intubated and resuscitated. Stabilised in ICU and remained in a vegetative state for 4 years
Kant et al. (1995) Neuropsychiatric institute, USA Case report Risk of bias: Y: 7 N: 1	**n:** 1 **Age:** 34 years **Gender:** Male **TBI severity:** Severe (GCS = 3) **Time post-injury:** 14 weeks **PTA:** RLAS, GOAT **Agitation:** Neurobehavioural Rating Scale	**Type:** ECT **Description:** Bilateral brief pulses (pulses > 25 s) **Setting:** Neuropsychiatric inpatient setting **Frequency:** 6 ECT treatments, three times per week **Duration:** 2 weeks	All psychotropic medications were discontinued prior to ECT (except Droperidol 2.5 mg once or twice daily for severe agitation)	**Primary: Agitation (qualitative)** Reduction in the frequency, duration and intensity of episodes of agitation **Primary: Harms** Mild confusion and worsening language deficits (cleared rapidly once course of ECT was complete) **Secondary: RLAS** Pre-intervention: 4; Post-intervention: 7 **Secondary: Sleep** Pre-intervention: 2–4 h fitful sleep; Post-intervention: 7–8 h uninterrupted sleep **Secondary: Cognitive functioning (MMSE)** Pre-intervention: 8; Post-intervention: 18; Follow-up: 22 **Secondary: Orientation (GOAT)** Pre-intervention: 5; Post-intervention: 67.5; Follow-up: 91.3 **Secondary: Cognition/energy (NRS)** Pre-intervention: 4.3; Post-intervention: 3.9; Follow-up: 2.4 **Secondary: Metacognition (NRS)** Pre-intervention: 5.0; Post-intervention: 3.3; Follow-up: 3.2 **Secondary: Somatic anxiety (NRS)** Pre-intervention: 4.3; Post-intervention: 2.8; Follow-up: 1.0 **Secondary: Language (NRS)** Pre-intervention: 4.5; Post-intervention: 2.5; Follow-up: 2.5

*ABC* antecedent-behaviour-consequence, *ABS* Agitated Behavior Scale, *bid* twice a day*, BNCE* Brief Neuropsychological Cognitive Examination, *CAM-ICU* Confusion Assessment Method for ICU, *CI* confidence interval, *CT* compliance training, *ECT* electroconvulsive therapy, *GOAT* Galveston Orientation and Amnesia Test, *GCS* Glasgow Coma Scale, *ICU* intensive care unit, *LoC* loss of consciousness, *M* mean, *MMSE* Mini-Mental State Examination, *n* number of participants, *NBP* neurobehavioural programme, *NRS* Neurobehavioural Rating Scale, *O-Log* Orientation Log, *PTA* post-traumatic amnesia, qhs *qd.* once a day, *qid* four times a day, *qhs* every bedtime*, RASS* Richmond Agitation-Sedation Scale, *RLAS* Rancho Los Amigos Scale*, SD* standard deviation, *TBI* traumatic brain injury, *tid* three times a day, *WPTAS* Westmead PTA Scale

#### Measurement of PTA

Formal measures of PTA status included the Westmead PTA Scale (WPTAS), Brief Neuropsychological Cognitive Examination (BNCE), Galveston Orientation and Amnesia Test (GOAT), Orientation Log (O-Log), Rancho Los Amigos Scale (RLAS) and Confusion Assessment Method for the ICU (CAM-ICU). Slifer et al. (1997) and Magee et al. (2011) reported formal assessment of PTA but did not describe the measure used. Formisano et al. (2001), Fluharty and Wallat (1997) and Berrol (1988) provided qualitative descriptors of PTA status.

#### Measurement of Agitation

Agitation was frequently measured in a non-standardised manner, which was a significant limitation of many included studies. Five studies used formal quantitative measurement tools to evaluate agitation, (namely the Agitated Behavior Scale [ABS] and Neurobehavioural Rating Scale), with only Park et al. (2016) and Baker (2001) providing pre- and post-intervention ABS scores. Other measurement tools used included a semi-quantitative scale of clinical modifications, direct behavioural observations, therapy attendance and agitated episodes requiring restraints and pharmacology.

#### Non-Pharmacological Interventions

Four studies examined a music intervention, five examined behaviour management and environmental modifications, two examined electroconvulsive therapy (ECT) and one examined physical restraint use. The music interventions examined live and taped music (Baker, 2001), preferred and classical relaxation music (Park et al., 2016), active, improvised music therapy (Formisano et al., 2001), and live familiar music (Magee et al., 2011). The duration of music exposure ranged from 5–60 min and was typically delivered at the bedside daily. The behaviour management and environmental modifications interventions included several strategies: a contingency/compliance protocol delivered by a 24-h behavioural assistant, close supervision (Slifer et al., 1996, 1997), distraction, time out, involvement in activities requiring full attention, embedding non-preferred activities, use of a locked ward (Fluharty & Wallat, 1997), reframing agitating stimuli, modifying ADLs to make them less demanding, desensitisation techniques, use of Premack principles (i.e., pairing high frequency behaviours with low frequency behaviours to increase cooperation), engagement in therapeutic activities (Fluharty & Glassman, 2001) and antecedent-behaviour-consequence (ABC) worksheets to address maladaptive cognitions and reduce agitation (Wilson et al., 2019). The ECT interventions were provided to patients who were refractory to pharmacological intervention; Nielsen et al. (2014) described bitemporal ECT, administered over three days, with 3-weekly maintenance treatments (ranging from five to eight sessions in total), whilst Kant et al. (1995) used bilateral-pulse ECT, administered three times per week for two weeks (six sessions in total). Physical restraint use described by Berrol (1988) included use of a vest and soft restraints; the relevant outcome for this study was harms, rather than agitation.

### Music Intervention

There were four studies involving music exposure or music therapy for reducing agitation. The two highest quality studies which used a randomised cross-over design and standardised measure of agitation (ABS) found that exposure to patient-preferred music, either live or taped, for 15–180 min per day, significantly reduced agitation when compared with no music (Baker, 2001) or classical relaxation music (Park et al., 2016). In one of these studies, patient-preferred music was also associated with a reduction in patient disorientation, indicating that music intervention may promote PTA recovery more broadly (Baker, 2001). The quality of these studies was compromised by the lack of blinding to treatment condition (e.g., music versus no music), small sample sizes, and a lack of power analysis, which makes it difficult to determine whether the findings represent a true effect. However, there was consistency in the benefits of preferred music on agitation across studies, including from a lower quality case series (Magee et al., 2011). Of note, 3/14 patients in the Park et al. (2016) study exhibited an unexpected increase in agitation with preferred music, which authors attributed to the genre of the music (i.e., music with a “strong beat and fast rhythm, such as heavy metal and rap”). This may suggest some constraints on the type of preferred music and the need to monitor outcomes to ensure a positive effect. One study examined music therapy and found that regular sessions of singing and instrument use were associated with improvement in psychomotor agitation, however, the quality of this study was low due to unstandardised assessment of agitation and no control condition to exclude natural recovery (Formisano et al., 2001).

### Behavioural and Environmental Strategies

Five studies reported implementing behavioural management strategies and environmental modifications for reducing agitation, although all suffered from significant methodological limitations. Using single case methods including a multiple baseline design, Slifer and colleagues found that the use of behavioural strategies (e.g., positive/negative reinforcement, ignoring disruptive behaviours, token economy) during daily therapy sessions, resulted in less agitation compared to baseline for a patient in PTA (Slifer et al., 1996). Slifer et al. (1997) found that a 24-h behavioural assistant who used behavioural strategies, along with a quiet environment, was also associated with a reduction in agitation during PTA. In two uncontrolled case studies, Fluharty and colleagues found that a combination of behavioural and environmental approaches (e.g., distraction, prompting, desensitisation, avoiding arguments, time out, engaging tasks, removing triggers and use of a locked ward), resulted in reduced incidents of aggressive, agitated or uncooperative behaviour from admission to discharge (Fluharty & Wallat, 1997; Fluharty & Glassman, 2001). All studies had significant flaws, in particular the absence of a control condition, thus it is difficult to exclude confounds such as natural recovery during PTA or the impact of concurrent interventions (e.g., pharmacological).

Using a more cognitively demanding approach, Wilson et al. (2019) found that a single session of cognitive-behaviour therapy (CBT) in which two patients in PTA were supported in addressing maladaptive cognitions contributing to agitated behaviours resulted in reduced agitation, and in one case improved anxiety and sleep. However, the absence of a control condition, lack of statistical analysis, and overall low quality of the study, limits any conclusions that can be drawn. Additionally, one of the cases was in the tail-end stages of PTA (unclear PTA stage for the other case) and it is difficult to see how patients deeper in PTA who often have higher levels of agitation (McKay et al., 2018), would be able to engage in a CBT-type of intervention.

### Physical Restraint

One case report described the use of a vest and soft restraints, along with mild sedation, with an agitated patient in early TBI recovery (Berrol, 1988). Changes in agitation were not assessed, although significant harms were reported. The patient was found unconscious and dangling from restraints, and whilst his condition eventually stabilised, he remained in a vegetative state for four years. This case report was alarming and stands alone as the only intervention in this review which reported on significant harm. However, the report lacked detail about agitation severity, the efficacy of the pharmacological intervention and whether restraints were necessary in the context of this behaviour. There was also little detail provided about the intervention used, such as how the restraints were applied and whether staff were trained in restraint use and supervision. This lack of detail makes it difficult to draw any convincing conclusions about the impact of restraint use on agitated patients in PTA. However, the study highlights some potential risks of restraint use that should be considered before use of such an intervention.

### Electroconvulsive Therapy

Two studies reported on the effectiveness of bilateral ECT for reducing agitation in patients experiencing delirium who were refractory to pharmacology and behavioural strategies (Kant et al., 1995; Nielsen et al., 2014). In both studies, agitation was noted to resolve following a course of ECT, measured quantitatively (using the RASS) and qualitatively. Cognitive functioning, orientation, and sleep were also noted to improve in one study (Kant et al., 1995). In terms of harms, mild confusion and worsening language deficits were reported by Kant et al. (1995) but this rapidly cleared following the course of ECT, although this change was not documented quantitatively. Other potential harms of ECT, such as sedation and cognitive decline, which could delay cognitive recovery, were not reported and consequently, it is difficult to evaluate whether ECT is a safe and appropriate intervention. Long-term follow-up would be important for ensuring ECT did not result in any lasting harms. In light of small sample sizes and a lack of methodological rigour, there is insufficient evidence to support the routine use of ECT to manage agitation in patients who are refractory to other interventions.

## Discussion

This systematic review synthesizes studies investigating the effectiveness of non-pharmacological intervention for managing agitation during PTA in adults with TBI. Twelve studies of low to moderate quality were included in the review. The non-pharmacological interventions were music therapy, environmental modifications and behavioural strategies, physical restraint and ECT. Overall, there was only weak evidence to support the effectiveness of non-pharmacological interventions for managing agitation during the PTA period after TBI.

Music intervention had the highest quality of evidence. All four studies reported that music intervention broadly reduced agitation levels during the PTA period, although benefits were limited to music reflective of pre-injury preferences as opposed to music that is generally considered calming (e.g., classical music). Preferred music may elicit positive memories and emotions, which in turn may reduce agitation in the same way that providing familiar photos or belongings may do (Park et al., 2016). Music selection may require adaptation for patients who prefer fast or heavy music, as this may increase agitation even if the music was preferred by the patient prior to their TBI (Park et al., 2016). It would be important to monitor patients’ agitation levels in response to preferred music genres, trial different music options and cease the intervention if there is persisting evidence of reduced tolerance or over-stimulation.

The same benefits were observed for taped music compared with live music performed by music therapists, which makes for an inexpensive and readily implementable intervention for agitation. Of course, it should be recognised that the music interventions were usually delivered in a systematic way in terms of duration and frequency (i.e., delivered daily for the same period of time), in limited doses with regular monitoring by the therapists involved. Additionally, these interventions were implemented bedside, which presumably minimised potential distractions. It could be argued that the one-to-one nature of the intervention, delivered in a quiet and controlled environment, and often involving human interaction, may contribute to the effectiveness of music in reducing agitated behaviours.

The longevity of benefit from music interventions on agitation levels is unclear. Park et al. (2016) suggested that there were no continuing effects of preferred music in the post-intervention phase, with agitation levels noted to increase at one hour post-intervention. This may be due to the short-term memory impairments characteristic of the PTA period, which would likely limit carryover of the intervention effects. Clinically, it may be important to consider how music therapy can be implemented in patients’ daily routine, such as during a less-preferred task or at a time when agitation is more severe, in order to maximise the calming effects of the intervention. This provides an interesting avenue for further research involving the delivery of music therapy.

Environmental and behavioural approaches are used frequently by clinicians worldwide to manage agitation during early TBI recovery (Carrier et al., 2021), and are recommended in expert guidelines (Ponsford et al., 2014). However, this review highlights the lack of empirical evidence to support these approaches. While behavioural approaches have a stronger evidence base in patients with TBI in the more chronic phase (Gould et al., 2021; Sloan, 2017; Ylvisaker et al., 2007), these may not translate to patients in PTA, given their significant cognitive impairments which can impact on the types of approaches that can be effective. In the studies included in this review, behavioural strategies often involved antecedent modification, where triggers of agitation are identified and removed or modified. For example, making changes to the environment, such as caring for someone in a quiet room if noise or other stimuli cause agitation, or using methods such as redirection for a person who is stuck on a topic that is causing agitation. Rewarding appropriate behaviours (e.g., using praise or a token economy) or ignoring disruptive behaviours was also highlighted.

Despite the limited evidence base for behavioural and environmental approaches, many of those that are recommended in expert guidelines can be plausibly implemented with little to no cost and minimal training, such as maintaining a quiet environment, allowing frequent rest periods, providing reassurance, and providing orienting or familiar information (Ponsford et al., 2014). Other approaches such as having a specialised environment, locked facilities or applying consistent behavioural principles using a 24h attendant will be more resource- and time-intensive and costly (Janzen et al., 2014; Mysiw & Sandel, 1997). Interventions involving active participation (such as CBT) may also be less feasible due to associated cognitive demands, such as the need for sustained attention and the ability to retain information across sessions. These interventions may also pose a risk of increasing agitation and may only be suitable for patients in the tail-end period of PTA, although at this stage, agitation is often resolved. Furthermore, in the absence of any control condition across studies, it is difficult to differentiate intervention effectiveness from natural recovery. Further research is needed to understand the most efficient and cost-effective methods for delivering environmental and behavioural interventions for a PTA population.

Physical restraint use was only explored in one case report, which highlighted the risk of significant harm associated with this intervention. As physical restraint use is not widely reported in the literature, it is difficult to draw conclusions about the effectiveness or harms of such an intervention for use with agitated patients. There may be limited reporting because restraint use results in adverse events such as those described by Berrol (1988). Further research is needed, given clinicians worldwide commonly use physical restraints for the management of agitation (Carrier et al., 2021), despite clinical guidelines recommending limited use due to risk of increased fear, confusion and agitation for patients in PTA (Ponsford et al., 2014). When restraints are used, Berrol (1988) highlights the need for strict guidelines including the need for regular monitoring. Ongoing staff training would be essential to ensure restraints are correctly administered in appropriate situations, consistent with research in other clinical populations which suggests staff education and training can significantly reduce the incidence of restraint use, thus reducing the risk of harms described in this review (Köpke et al., 2012; Testad et al., 2005). Environmental modifications, such as a lowered bed with padded walls, should also be considered as alternatives to restraint use (Ponsford et al., 2014).

The effect of ECT on agitation after TBI was reported in a case series and case report, both which found that bilateral brief-pulse ECT reduced agitation in patients who were refractory to other treatments. Despite the reported efficacy, there was a lack of standardised outcome measurement and neither study conducted thorough cognitive testing to determine potential side effects of the ECT treatment. Evidence of mild confusion and worsening language deficits were noted clinically (Kant et al., 1995), although it is unclear whether this reflected a side effect of ECT or fluctuations in cognition common in PTA. Electroconvulsive therapy is an invasive procedure, with well-documented risks (Ingram et al., 2008); it is unlikely to become a routine component of agitation management in TBI care settings. Furthermore, TBI patients are frequently prescribed a range of medications (such as anticonvulsants) to manage multiple injuries and comorbidities, which may have interaction effects when combined with ECT. These factors need to be carefully considered, along with the side effect profile of ECT, in determining the suitability of this intervention. There is a need for more controlled evidence to support ECT as a possible alternative intervention for patients experiencing significant agitation, who have no contraindications to ECT and have proved refractory to other interventions.

### Summary of Findings and Study Limitations

The findings of this review highlight the lack of research examining non-pharmacological management of agitation during the PTA period following TBI. This is problematic given expert recommendations support the use of non-pharmacological intervention as a first-line approach, and pharmacological interventions are thought to have limited efficacy and potential adverse effects (Hicks et al., 2018; McKay et al., 2021; Mehta et al., 2018; Nash et al., 2019; Ponsford et al., 2014; Williamson et al., 2019). Only four of the 12 included studies were published in the last decade, indicating a decline in research on this topic. Furthermore, the range of non-pharmacological interventions identified was limited, despite the broad scope of the review. For example, there was an absence of research relating to staff training and education, despite the integral role of staff in the management of agitated behaviours. There are various factors that may contribute to stagnation in this research area, including the heterogenous nature of TBI and the transient nature of PTA, which makes studying and managing this population challenging. The evaluation of non-pharmacological strategies should be an important consideration in future research; and pharmacological studies should consider systematically monitoring concomitant non-pharmacological strategies used, given clinical management of agitation often involves implementing a combination of these strategies.

Across most studies, it was difficult to delineate whether improvement in agitation was a result of the intervention or reflective of natural recovery due to the absence of a control condition. In addition, most of the existing studies exploring non-pharmacological management of agitation are limited by small sample sizes and methodological flaws. For example, most studies lacked a formalised measure of agitation. Validated measurement tools (such as the ABS) and consistent nomenclature are important for the consistent measurement of agitation, which would improve the quality of studies published in this area. Consistent agitation measurement and nomenclature are also important practices in clinical settings, particularly for identifying potential triggers for agitation, determining the clinical effectiveness of interventions implemented, and facilitating effective communication among clinicians and family members (Fugate et al., 1997; Janzen et al., 2014). Additionally, many studies did not control for the concomitant use of pharmacological interventions; medication was rarely regulated or limited during the intervention period, and often poorly described. This limits the ability to draw any conclusions about the effectiveness of the non-pharmacological interventions under investigation. Additionally, many studies did not describe time post-injury, which makes it difficult to generalise these findings, as agitation levels can fluctuate according to the level of cognitive recovery, and often in a non-linear manner (McKay et al., 2018). Finally, the absence of follow-up procedures in many of the included studies made it difficult to determine the lasting impact of the described interventions. Overall, future studies should aim to use a formal measure of agitation, control for concomitant interventions and natural recovery, describe the stage of recovery in sufficient detail and conduct suitable follow-up procedures.

It is important to consider interventions examined in excluded studies that assessed agitation as a secondary outcome. Several interventions monitored agitation as a means of determining patient tolerance to a particular intervention during PTA (i.e., not increasing agitation rather than actively reducing agitation), as agitation is a common barrier to rehabilitation. For example, several studies found virtual reality may be an effective intervention for improving attention during PTA, with limited risk of increasing agitation (Dvorkin et al., 2009; Larson et al., 2011). Similarly, Trevena-Peters et al. (2018a) found that activities of daily living (ADL) retraining may improve functional independence in patients in PTA without increasing agitation during this period. Understanding the interventions that do not risk increasing agitation is important for ensuring patients in PTA receive appropriate stimulation for their stage of recovery. This is pertinent in the context of findings by Trevena-Peters et al. (2018b), which suggest that early intervention may improve rehabilitation outcomes despite cognitive limitations associated with PTA. Furthermore, interventions tolerated during PTA could be combined with successful non-pharmacological strategies for reducing agitation discussed here (e.g., virtual reality delivered in a calm and quiet environment) to improve the likelihood of intervention success.

### Review Limitations

The search strategy was restricted to studies published in English. However, there were several eligible English abstracts with full texts provided in a language other than English. For these studies, the full text was translated to confirm eligibility, which may have reduced the impact of this bias on the review. In terms of addressing publication bias, the search strategy included a comprehensive search of unpublished studies through searching of clinical trial registries and contact with key authors in the field. Several studies were excluded because they involved participants who were no longer in PTA or had been discharged to an outpatient setting. These studies were excluded as the agitated behaviours were more chronic in nature (given time since injury) and are reflective of a smaller subset of individuals who experience ongoing neurobehavioural problems. To our knowledge, this review represents the only systematic review of the evidence for non-pharmacological interventions for agitation during PTA after TBI. It examined both the efficacy and harms of non-pharmacological interventions, employed a comprehensive strategy and rigorous analysis of methodological quality.

### Conclusions

Agitated behaviours are one of the most significant and disruptive sequalae exhibited during PTA and it is evident that researchers and clinicians worldwide are grappling with the challenges of managing agitation (Bogner et al., 2001; Carrier et al., 2021; Kadyan et al., 2004; Nott et al., 2006). Music therapy had the highest quality of evidence, with preferred music in taped or live format showing promise in reducing agitation. Behavioural and environmental strategies (such as contingency management, antecedent modification, distraction, and positive reinforcement) may assist in reducing agitation, particularly where the approach is flexible and patient specific, although methodological limitations of the existing research mean the true efficacy of these approaches remains unclear. The harms of physical restraints were highlighted, which support recommendations to avoid restraint use where possible (Ponsford et al., 2014). The use of ECT for patients refractory to pharmacological and behavioural interventions was also described, although given the significant associated risks, which included a potential negative impact on memory, this approach is not recommended. Overall, randomised controlled trials with inclusion of a control group and use of a formal measurement tool for assessing agitation are a critical next step in developing suitable recommendations for the effective non-pharmacological management of agitation after TBI.

## Supplementary Information

Below is the link to the electronic supplementary material.

Supplementary file1 (PDF 1851 KB)

Supplementary file2 (DOCX 33 KB)

Supplementary file3 (DOCX 39 KB)

Supplementary file4 (DOCX 19 KB)

Supplementary file5 (DOCX 77 KB)

## Data Availability

The full systematic review search strategy used in this review is provided in Online Resource 2.
