# Supplementary material for: Effectiveness of Non-Pharmacological Interventions for Agitation during Post-Traumatic Amnesia following Traumatic Brain Injury: A Systematic Review
Source: Neuropsychol Rev. 2022 Jun 10;33(2):374–92. doi: 10.1007/s11065-022-09544-5 (PMC10148768; doi:10.1007/s11065-022-09544-5)
Supplement: Supplementary file 2 — Supplementary file2 (DOCX 33 KB) [file 11065_2022_9544_MOESM2_ESM.docx]

**Online Resource 2: Systematic Review Search Strategy**

**A. CINAHL Plus with Full Text**

Limiters - Exclude MEDLINE records

( ( (MH "Intracranial Hemorrhage+") OR (MH "Brain Injuries+") OR (MH "Cerebral Hemorrhage+") OR (MH "Head Injuries") OR (MH "Subarachnoid Hemorrhage") OR (MH "Brain Damage, Chronic+") OR (MH "Pneumocephalus") ) OR TI ( ((Brain OR Cerebr* OR "Contre Coup" OR Contrecoup OR Crani* OR Crushing Skull OR Diffuse Axonal OR Head OR Hemisphere* OR Skull) N1 (Injur* OR Trauma*)) OR ((Cerebr* OR Crani* OR Head) N1 (Lesion* OR Wound*)) OR ((Brain* OR Cereb* OR Cortical) N1 Contusion*) OR "Brain Damage" OR Concuss* OR DAI OR DAIs OR Impact Seizure* OR Pneumocephalus OR ((Traumatic OR Posttraumatic) N1 (Brain* OR Cerebr* OR Coma* OR Encephalopath* OR Epilep* OR Intracerebral OR Midbrain OR Seizure* OR Subarachnoid)) OR ((Forehead OR Frontal OR Frontal Region OR Occipital OR Occipital Region OR Parietal OR Parietal Region OR Temporal OR Temporal Region) N1 (Trauma* OR Posttrauma*)) OR TBI OR TBIs ) OR AB ( ((Brain OR Cerebr* OR "Contre Coup" OR Contrecoup OR Crani* OR Crushing Skull OR Diffuse Axonal OR Head OR Hemisphere* OR Skull) N1 (Injur* OR Trauma*)) OR ((Cerebr* OR Crani* OR Head) N1 (Lesion* OR Wound*)) OR ((Brain* OR Cereb* OR Cortical) N1 Contusion*) OR "Brain Damage" OR Concuss* OR DAI OR DAIs OR Impact Seizure* OR Pneumocephalus OR ((Traumatic OR Posttraumatic) N1 (Brain* OR Cerebr* OR Coma* OR Encephalopath* OR Epilep* OR Intracerebral OR Midbrain OR Seizure* OR Subarachnoid)) OR ((Forehead OR Frontal OR Frontal Region OR Occipital OR Occipital Region OR Parietal OR Parietal Region OR Temporal OR Temporal Region) N1 (Trauma* OR Posttrauma*)) OR TBI OR TBIs ) ) AND ( ( (MH "Aggression") OR (MH "Violence") OR (MH "Assertiveness") OR (MH "Self-Injurious Behavior") OR (MH "Impulsive Behavior+") OR (MH "Compulsive Behavior") OR (MH "Wandering Behavior") OR (MH "Imitative Behavior") OR (MH "Anger") OR (MH "Acting Out") OR (MH "Agitation") OR (MH "Psychomotor Agitation") OR (MH "Restraint, Physical") OR (MH "Physical Restraint (Iowa NIC)") OR (MH "Patient Safety") OR (MH "Hallucinations") OR (MH "Delusions") OR (MH "Paranoid Disorders") OR (MH "Patient Compliance") OR (MH "Attention") OR (MH "Suicide, Attempted") ) OR TI ( Abus* OR Abscond* OR "Act Out" OR "Acted Out" OR "Acting Out" OR "Acts Out" OR Assertive* OR Behavio#r* of Concern OR Challenging Behavio#r* OR Concerning Behavio#r* OR Repetitive Behavio#r* OR Stereotyp* OR Stimulating Behavio#r* OR Frustrat* OR Hallucinat* OR Delusion* OR Paranoi* OR Rancho* OR "Self Destructive" OR "Self Harm" OR "Self Injuries" OR "Self Injurious" OR "Self Injury" OR Acathisia* OR Adheren* OR Complian* OR Cooperat* OR Nonadheren* OR Noncomplian* OR Aggress* OR Agitat* OR Anger# OR Angry OR Akathisia* OR Compuls* OR Demanding OR Disinhib* OR Insight* OR Excit* OR Excessive OR Hyperactiv* OR Impuls* OR Impatien* OR Intoleran* OR Toleran* OR Wander* OR Attention OR Inattention OR Concentrat* OR Distract* OR Unpredictab* OR Overt Behavio* OR OBS OR Oppos* OR Parasuicide* OR Attempted Suicide* OR PAS OR RASS OR Resistan* OR Restless* OR RLAS OR Restrain* OR Immobiliz* OR Immobilis* OR Seclu* OR Safety OR Threaten* OR Violen* OR Verbal Preservation OR Motor Preservation OR Emotional Lability ) OR AB ( Abus* OR Abscond* OR "Act Out" OR "Acted Out" OR "Acting Out" OR "Acts Out" OR Assertive* OR Behavio#r* of Concern OR Challenging Behavio#r* OR Concerning Behavio#r* OR Repetitive Behavio#r* OR Stereotyp* OR Stimulating Behavio#r* OR Frustrat* OR Hallucinat* OR Delusion* OR Paranoi* OR Rancho* OR "Self Destructive" OR "Self Harm" OR "Self Injuries" OR "Self Injurious" OR "Self Injury" OR Acathisia* OR Adheren* OR Complian* OR Cooperat* OR Nonadheren* OR Noncomplian* OR Aggress* OR Agitat* OR Anger# OR Angry OR Akathisia* OR Compuls* OR Demanding OR Disinhib* OR Insight* OR Excit* OR Excessive OR Hyperactiv* OR Impuls* OR Impatien* OR Intoleran* OR Toleran* OR Wander* OR Attention OR Inattention OR Concentrat* OR Distract* OR Unpredictab* OR Overt Behavio* OR OBS OR Oppos* OR Parasuicide* OR Attempted Suicide* OR PAS OR RASS OR Resistan* OR Restless* OR RLAS OR Restrain* OR Immobiliz* OR Immobilis* OR Seclu* OR Safety OR Threaten* OR Violen* OR Verbal Preservation OR Motor Preservation OR Emotional Lability ) ) AND ( ( (MH "Memory Disorders") OR (MH "Amnesia") OR (MH "Confabulation") OR (MH "Delirium") OR (MH "Confusion+") OR (MH "Orientation") ) OR TI ( Amnes* OR "Cognitive Retention Disorder" OR "Cognitive Retention Disorders" OR "Memory Deficit" OR "Memory Deficits" OR "Memory Disorder" OR "Memory Disorders" OR "Memory Loss" OR "Memory Losses" OR Rancho* OR "Retention Disorder" OR "Retention Disorders" OR RLAS OR Confabulat* OR Dysmnesic OR Disorientat* OR GOAT OR O-Log OR Orientation* OR PTA OR Confus* OR PTCS OR Delirium* OR WPTAS* ) OR TI ( Amnes* OR "Cognitive Retention Disorder" OR "Cognitive Retention Disorders" OR "Memory Deficit" OR "Memory Deficits" OR "Memory Disorder" OR "Memory Disorders" OR "Memory Loss" OR "Memory Losses" OR Rancho* OR "Retention Disorder" OR "Retention Disorders" OR RLAS OR Confabulat* OR Dysmnesic OR Disorientat* OR GOAT OR O-Log OR Orientation* OR PTA OR Confus* OR PTCS OR Delirium* OR WPTAS* ) )

**B. ClinicalTrials.Gov**

Disease or Condition: (Trauma OR Traumatic OR Posttrauma OR Posttraumatic OR TBI) AND (Aggression OR Violence OR Agitation OR Self-Injury OR Impulsive OR Compulsive OR Hallucination OR Angry OR Amnesia OR Memory OR Delirium OR Confusion OR Orientation)

**C. Cochrane Database of Systematic Reviews (CDSR) and Cochrane Central Regiter of Controlled Trials (CENTRAL)**

([mh "Brain Hemorrhage, Traumatic"] OR [mh "Brain Injuries"] OR [mh "Brain Injuries, Traumatic"] OR [mh "Brain Stem Hemorrhage, Traumatic"] OR [mh "Cerebral Hemorrhage, Traumatic"] OR [mh ^"Craniocerebral Trauma"] OR [mh "Epilepsy, Post-Traumatic"] OR [mh "Head Injuries, Closed"] OR [mh "Head Injuries, Penetrating"] OR [mh "Intracranial Hemorrhage, Traumatic"] OR [mh "Subarachnoid Hemorrhage, Traumatic"] OR [mh "Brain Damage, Chronic"] OR [mh "Coma, Post-Head Injury"] OR [mh "Brain Injury, Chronic"] OR [mh ^"Cerebrovascular Trauma"] OR [mh "Diffuse Axonal Injury"] OR [mh Pneumocephalus] OR ((Brain OR Cerebr* OR "Contre Coup" OR Contrecoup OR Crani* OR Crushing Skull OR Diffuse Axonal OR Head OR Hemisphere* OR Skull) NEAR/1 (Injur* OR Trauma*)) OR ((Cerebr* OR Crani* OR Head) NEAR/1 (Lesion* OR Wound*)) OR ((Brain* OR Cereb* OR Cortical) NEAR/1 Contusion*) OR "Brain Damage" OR Concuss* OR DAI OR DAIs OR Impact Seizure* OR Pneumocephalus OR ((Traumatic OR Posttraumatic) NEAR/1 (Brain* OR Cerebr* OR Coma* OR Encephalopath* OR Epilep* OR Intracerebral OR Midbrain OR Seizure* OR Subarachnoid)) OR ((Forehead OR Frontal OR Frontal Region OR Occipital OR Occipital Region OR Parietal OR Parietal Region OR Temporal OR Temporal Region) NEAR/1 (Trauma* OR Posttrauma*)) OR TBI OR TBIs) AND ([mh ^Aggression] OR [mh ^Violence] OR [mh ^"Psychomotor Agitation"] OR [mh "Acting Out"] OR [mh ^"Self-Injurious Behavior"] OR [mh ^"Impulsive Behavior"] OR [mh ^"Restraint, Physical"] OR [mh "Patient Safety"] OR [mh ^"Safety Management"] OR [mh ^Hallucinations] OR [mh Delusions] OR [mh "Paranoid Behavior"] OR [mh ^"Patient Compliance"] OR [mh ^Anger] OR [mh "Wandering Behavior"] OR [mh Assertiveness] OR [mh ^Attention] OR [mh "Suicide, Attempted"] OR [mh ^"Compulsive Behavior"] OR Abus* OR Abscond* OR "Act Out" OR "Acted Out" OR "Acting Out" OR "Acts Out" OR Assertive* OR "Behavio?r* of Concern" OR Challenging Behavio?r* OR Concerning Behavio?r* OR Repetitive Behavio?r* OR Stereotyp* OR Stimulating Behavio?r* OR Frustrat* OR Hallucinat* OR Delusion* OR Paranoi* OR Rancho* OR "Self Destructive" OR "Self Harm" OR "Self Injuries" OR "Self Injurious" OR "Self Injury" OR Acathisia* OR Adheren* OR Complian* OR Cooperat* OR Nonadheren* OR Noncomplian* OR Aggress* OR Agitat* OR Anger? OR Angry OR Akathisia* OR Compuls* OR Demanding OR Disinhib* OR Insight* OR Excit* OR Excessive OR Hyperactiv* OR Impuls* OR Impatien* OR Intoleran* OR Toleran* OR Wander* OR Attention OR Inattention OR Concentrat* OR Distract* OR Unpredictab* OR Overt Behavio* OR OBS OR Oppos* OR Parasuicide* OR Attempted Suicide* OR PAS OR RASS OR Resistan* OR Restless* OR RLAS OR Restrain* OR Immobiliz* OR Immobilis* OR Seclu* OR Safety OR Threaten* OR Violen* OR Verbal Preservation OR Motor Preservation OR Emotional Lability) AND ([mh "Amnesia"] OR [mh "Memory Disorders"] OR [mh "Delirium"] OR [mh "Confusion"] OR [mh "Orientation"] OR Amnes* OR "Cognitive Retention Disorder" OR "Cognitive Retention Disorders" OR "Memory Deficit" OR "Memory Deficits" OR "Memory Disorder" OR "Memory Disorders" OR "Memory Loss" OR "Memory Losses" OR Rancho* OR "Retention Disorder" OR "Retention Disorders" OR RLAS OR Confabulat* OR Dysmnesic OR Disorientat* OR GOAT OR O-Log OR Orientation* OR PTA OR Confus* OR PTCS OR Delirium* OR WPTAS*)

Limits: in Cochrane Reviews, Cochrane Protocols and Trials

**D. Embase 1974 to May 2020**

1. *Brain Hemorrhage/ OR Exp *Brain Injury/ OR *Head Injury/ OR *Subarachnoid Hemorrhage/ OR *Traumatic Epilepsy/ OR (((Brain OR Cerebr* OR "Contre Coup" OR Contrecoup OR Crani* OR Crushing Skull OR Diffuse Axonal OR Head OR Hemisphere* OR Skull) adj1 (Injur* OR Trauma*)) OR ((Cerebr* OR Crani* OR Head) adj1 (Lesion* OR Wound*)) OR ((Brain* OR Cereb* OR Cortical) adj1 Contusion*) OR "Brain Damage" OR Concuss* OR DAI OR DAIs OR Impact Seizure* OR Pneumocephalus OR ((Traumatic OR Posttraumatic) adj1 (Brain* OR Cerebr* OR Coma* OR Encephalopath* OR Epilep* OR Intracerebral OR Midbrain OR Seizure* OR Subarachnoid)) OR ((Forehead OR Frontal OR Frontal Region OR Occipital OR Occipital Region OR Parietal OR Parietal Region OR Temporal OR Temporal Region) adj1 (Trauma* OR Posttrauma*)) OR TBI OR TBIs).mp.
2. *Aggression/ OR *Agitation/ OR Exp *Agitation Assessment/ OR *Violence/ OR Exp *Acting Out/ OR *Impulsiveness/ OR Exp *Compulsion/ OR *Restlessness/ OR Exp *Automutilation/ OR *Akathisia/ OR *Patient Safety/ OR *Safety/ OR Exp *Hallucination/ OR Exp *Delusion/ OR *Paranoia/ OR *Patient Compliance/ OR *Anger/ OR *State-Trait Anger Expression Inventory/ OR *Wandering Behavior/ OR *Assertiveness/ OR *Attention/ OR *Mental Concentration/ OR *Distractibility/ OR *Suicide Attempt/ OR (Abus* OR Abscond* OR "Act Out" OR "Acted Out" OR "Acting Out" OR "Acts Out" OR Assertive* OR Behavio?r* of Concern OR Challenging Behavio?r* OR Concerning Behavio?r* OR Repetitive Behavio?r* OR Stereotyp* OR Stimulating Behavio?r* OR Frustrat* OR Hallucinat* OR Delusion* OR Paranoi* OR Rancho* OR "Self Destructive" OR "Self Harm" OR "Self Injuries" OR "Self Injurious" OR "Self Injury" OR Acathisia* OR Adheren* OR Complian* OR Cooperat* OR Nonadheren* OR Noncomplian* OR Aggress* OR Agitat* OR Anger? OR Angry OR Akathisia* OR Compuls* OR Demanding OR Disinhib* OR Insight* OR Excit* OR Excessive OR Hyperactiv* OR Impuls* OR Impatien* OR Intoleran* OR Toleran* OR Wander* OR Attention OR Inattention OR Concentrat* OR Distract* OR Unpredictab* OR Overt Behavio* OR OBS OR Oppos* OR Parasuicide* OR Attempted Suicide* OR PAS OR RASS OR Resistan* OR Restless* OR RLAS OR Restrain* OR Immobiliz* OR Immobilis* OR Seclu* OR Safety OR Threaten* OR Violen* OR Verbal Preservation OR Motor Preservation OR Emotional Lability).mp.
3. Exp *Amnesia/ OR Exp *Delirium/ OR Exp *Confusion/ OR Exp *Orientation/ OR Exp *Memory Disorder/ OR (Amnes* OR "Cognitive Retention Disorder" OR "Cognitive Retention Disorders" OR "Memory Deficit" OR "Memory Deficits" OR "Memory Disorder" OR "Memory Disorders" OR "Memory Loss" OR "Memory Losses" OR Rancho* OR "Retention Disorder" OR "Retention Disorders" OR RLAS OR Confabulat* OR Dysmnesic OR Disorientat* OR GOAT OR O-Log OR Orientation* OR PTA OR Confus* OR PTCS OR Delirium* OR WPTAS*).mp.
4. 1 AND 2 AND 3
5. Exp Animals/ OR Exp Invertebrate/ OR Animal Experiment/ OR Animal Model/ OR Animal Tissue/ OR Animal Cell/ OR Nonhuman/
6. Human/ OR Normal Human/ OR Human Cell/
7. 5 AND 6
8. 5 NOT 7
9. 4 NOT 8
10. Limit 9 to EMBASE

**E. Ovid MEDLINE(R) ALL 1946 to May 2020**

1. Exp Brain Hemorrhage, Traumatic/ OR Exp Brain Injuries/ OR Exp Brain Injuries, Traumatic/ OR Exp Brain Stem Hemorrhage, Traumatic/ OR Exp Cerebral Hemorrhage, Traumatic/ OR Craniocerebral Trauma/ OR Exp Epilepsy, Post-Traumatic/ OR Exp Head Injuries, Closed/ OR Exp Head Injuries, Penetrating/ OR Exp Intracranial Hemorrhage, Traumatic/ OR Exp Subarachnoid Hemorrhage, Traumatic/ OR Exp Brain Damage, Chronic/ OR Exp Coma, Post-Head Injury/ OR Brain Injury, Chronic/ OR Cerebrovascular Trauma/ OR Diffuse Axonal Injury/ OR Exp Pneumocephalus/ OR (((Brain OR Cerebr* OR "Contre Coup" OR Contrecoup OR Crani* OR Crushing Skull OR Diffuse Axonal OR Head OR Hemisphere* OR Skull) adj1 (Injur* OR Trauma*)) OR ((Cerebr* OR Crani* OR Head) adj1 (Lesion* OR Wound*)) OR ((Brain* OR Cereb* OR Cortical) adj1 Contusion*) OR "Brain Damage" OR Concuss* OR DAI OR DAIs OR Impact Seizure* OR Pneumocephalus OR ((Traumatic OR Posttraumatic) adj1 (Brain* OR Cerebr* OR Coma* OR Encephalopath* OR Epilep* OR Intracerebral OR Midbrain OR Seizure* OR Subarachnoid)) OR ((Forehead OR Frontal OR Frontal Region OR Occipital OR Occipital Region OR Parietal OR Parietal Region OR Temporal OR Temporal Region) adj1 (Trauma* OR Posttrauma*)) OR TBI OR TBIs).mp.
2. Aggression/ OR Violence/ OR Exp Psychomotor Agitation/ OR Exp Acting Out/ OR Exp Self-Injurious Behavior/ OR Impulsive Behavior/ OR Exp Compulsive Behavior/ OR Restraint, Physical/ OR Patient Safety/ OR Safety Management/ OR Hallucinations/ OR Delusions/ OR Paranoid Behavior/ OR Patient Compliance/ OR Anger/ OR Wandering Behavior/ OR Assertiveness/ OR Attention/ OR Suicide, Attempted/ OR (Abus* OR Abscond* OR "Act Out" OR "Acted Out" OR "Acting Out" OR "Acts Out" OR Assertive* OR Behavio?r* of Concern OR Challenging Behavio?r* OR Concerning Behavio?r* OR Repetitive Behavio?r* OR Stereotyp* OR Stimulating Behavio?r* OR Frustrat* OR Hallucinat* OR Delusion* OR Paranoi* OR Rancho* OR "Self Destructive" OR "Self Harm" OR "Self Injuries" OR "Self Injurious" OR "Self Injury" OR Acathisia* OR Adheren* OR Complian* OR Cooperat* OR Nonadheren* OR Noncomplian* OR Aggress* OR Agitat* OR Anger? OR Angry OR Akathisia* OR Compuls* OR Demanding OR Disinhib* OR Insight* OR Excit* OR Excessive OR Hyperactiv* OR Impuls* OR Impatien* OR Intoleran* OR Toleran* OR Wander* OR Attention OR Inattention OR Concentrat* OR Distract* OR Unpredictab* OR Overt Behavio* OR OBS OR Oppos* OR Parasuicide* OR Attempted Suicide* OR PAS OR RASS OR Resistan* OR Restless* OR RLAS OR Restrain* OR Immobiliz* OR Immobilis* OR Seclu* OR Safety OR Threaten* OR Violen* OR Verbal Preservation OR Motor Preservation OR Emotional Lability).mp.
3. Exp Amnesia/ OR Exp Memory Disorders/ OR Exp Delirium/ OR Exp Confusion/ OR Exp Orientation/ OR (Amnes* OR "Cognitive Retention Disorder" OR "Cognitive Retention Disorders" OR "Memory Deficit" OR "Memory Deficits" OR "Memory Disorder" OR "Memory Disorders" OR "Memory Loss" OR "Memory Losses" OR Rancho* OR "Retention Disorder" OR "Retention Disorders" OR RLAS OR Confabulat* OR Dysmnesic OR Disorientat* OR GOAT OR O-Log OR Orientation* OR PTA OR Confus* OR PTCS OR Delirium* OR WPTAS*).mp.
4. 1 AND 2 AND 3
5. Exp Animals/ NOT Humans.sh.
6. 4 NOT 5

**F. APA PsycInfo 1806 to May 2020**

1. Brain Damage/ OR Cerebral Hemorrhage/ OR Exp Head Injuries/ OR Subarachnoid Hemorrhage/ OR Exp Traumatic Brain Injury/ OR (((Brain OR Cerebr* OR "Contre Coup" OR Contrecoup OR Crani* OR Crushing Skull OR Diffuse Axonal OR Head OR Hemisphere* OR Skull) adj1 (Injur* OR Trauma*)) OR ((Cerebr* OR Crani* OR Head) adj1 (Lesion* OR Wound*)) OR ((Brain* OR Cereb* OR Cortical) adj1 Contusion*) OR "Brain Damage" OR Concuss* OR DAI OR DAIs OR Impact Seizure* OR Pneumocephalus OR ((Traumatic OR Posttraumatic) adj1 (Brain* OR Cerebr* OR Coma* OR Encephalopath* OR Epilep* OR Intracerebral OR Midbrain OR Seizure* OR Subarachnoid)) OR ((Forehead OR Frontal OR Frontal Region OR Occipital OR Occipital Region OR Parietal OR Parietal Region OR Temporal OR Temporal Region) adj1 (Trauma* OR Posttrauma*)) OR TBI OR TBIs).mp.
2. Aggressive Behavior/ OR Aggressiveness/ OR Agitation/ OR Exp Acting Out/ OR Exp Self-Injurious Behavior/ OR Impulsiveness/ OR Exp Compulsions/ OR Akathisia/ OR Restlessness/ OR (Abus* OR Abscond* OR "Act Out" OR "Acted Out" OR "Acting Out" OR "Acts Out" OR Assertive* OR Behavio?r* of Concern OR Challenging Behavio?r* OR Concerning Behavio?r* OR Repetitive Behavio?r* OR Stereotyp* OR Stimulating Behavio?r* OR Frustrat* OR Hallucinat* OR Delusion* OR Paranoi* OR Rancho* OR "Self Destructive" OR "Self Harm" OR "Self Injuries" OR "Self Injurious" OR "Self Injury" OR Acathisia* OR Adheren* OR Complian* OR Cooperat* OR Nonadheren* OR Noncomplian* OR Aggress* OR Agitat* OR Anger? OR Angry OR Akathisia* OR Compuls* OR Demanding OR Disinhib* OR Insight* OR Excit* OR Excessive OR Hyperactiv* OR Impuls* OR Impatien* OR Intoleran* OR Toleran* OR Wander* OR Attention OR Inattention OR Concentrat* OR Distract* OR Unpredictab* OR Overt Behavio* OR OBS OR Oppos* OR Parasuicide* OR Attempted Suicide* OR PAS OR RASS OR Resistan* OR Restless* OR RLAS OR Restrain* OR Immobiliz* OR Immobilis* OR Seclu* OR Safety OR Threaten* OR Violen* OR Verbal Preservation OR Motor Preservation OR Emotional Lability).mp.
3. Exp Amnesia/ OR Exp Delirium/ OR Delirium Tremens/ OR Exp Mental Confusion/ OR Exp Perceptual Orientation/ OR Exp "Memory Disorders"/ OR (Amnes* OR "Cognitive Retention Disorder" OR "Cognitive Retention Disorders" OR "Memory Deficit" OR "Memory Deficits" OR "Memory Disorder" OR "Memory Disorders" OR "Memory Loss" OR "Memory Losses" OR Rancho* OR "Retention Disorder" OR "Retention Disorders" OR RLAS OR Confabulat* OR Dysmnesic OR Disorientat* OR GOAT OR O-Log OR Orientation* OR PTA OR Confus* OR PTCS OR Delirium* OR WPTAS*).mp.
4. 1 AND 2 AND 3

**G. PubMed**

("Brain Hemorrhage, Traumatic"[mh] OR "Brain Injuries"[mh] OR "Brain Injuries, Traumatic"[mh] OR "Brain Stem Hemorrhage, Traumatic"[mh] OR "Cerebral Hemorrhage, Traumatic"[mh] OR "Craniocerebral Trauma"[mh:noexp] OR "Epilepsy, Post-Traumatic"[mh] OR "Head Injuries, Closed"[mh] OR "Head Injuries, Penetrating"[mh] OR "Intracranial Hemorrhage, Traumatic"[mh] OR "Subarachnoid Hemorrhage, Traumatic"[mh] OR "Brain Damage, Chronic"[mh] OR "Coma, Post-Head Injury"[mh] OR "Brain Injury, Chronic"[mh] OR "Cerebrovascular Trauma"[mh:noexp] OR "Diffuse Axonal Injury"[mh] OR Pneumocephalus[mh] OR ((Brain OR Cerebr* OR "Contre Coup" OR Contrecoup OR Crani* OR Crushing Skull OR Diffuse Axonal OR Head OR Hemisphere* OR Skull) AND (Injur* OR Trauma*)) OR ((Cerebr* OR Crani* OR Head) AND (Lesion* OR Wound*)) OR ((Brain* OR Cereb* OR Cortical) AND Contusion*) OR "Brain Damage" OR Concuss* OR DAI OR DAIs OR Impact Seizure* OR Pneumocephalus OR ((Traumatic OR Posttraumatic) AND (Brain* OR Cerebr* OR Coma* OR Encephalopath* OR Epilep* OR Intracerebral OR Midbrain OR Seizure* OR Subarachnoid)) OR ((Forehead OR Frontal OR Frontal Region OR Occipital OR Occipital Region OR Parietal OR Parietal Region OR Temporal OR Temporal Region) AND (Trauma* OR Posttrauma*)) OR TBI OR TBIs) AND (Aggression[mh:noexp] OR Violence[mh:noexp] OR "Psychomotor Agitation"[mh:noexp] OR "Acting Out"[mh] OR "Self-Injurious Behavior"[mh:noexp] OR "Impulsive Behavior"[mh:noexp] OR "Restraint, Physical"[mh:noexp] OR "Patient Safety"[mh] OR "Safety Management"[mh:noexp] OR Hallucinations[mh:noexp] OR Delusions[mh] OR "Paranoid Behavior"[mh] OR "Patient Compliance"[mh:noexp] OR Anger[mh:noexp] OR "Wandering Behavior"[mh] OR Assertiveness[mh] OR Attention[mh:noexp] OR "Suicide, Attempted"[mh] OR "Compulsive Behavior"[mh:noexp] OR Abus* OR Abscond* OR "Act Out" OR "Acted Out" OR "Acting Out" OR Assertive* OR "Behaviour of Concern" OR Challenging Behavi* OR Concerning Behavi* OR Repetitive Behavi* OR Stereotyp* OR Stimulating Behavi* OR Frustrat* OR Hallucinat* OR Delusion* OR Paranoi* OR Rancho* OR "Self Destructive" OR "Self Harm" OR "Self Injuries" OR "Self Injurious" OR "Self Injury" OR Acathisia* OR Adheren* OR Complian* OR Cooperat* OR Nonadheren* OR Noncomplian* OR Aggress* OR Agitat* OR Anger* OR Angry OR Akathisia* OR Compuls* OR Demanding OR Disinhib* OR Insight* OR Excit* OR Excessive OR Hyperactiv* OR Impuls* OR Impatien* OR Intoleran* OR Toleran* OR Wander* OR Attention OR Inattention OR Concentrat* OR Distract* OR Unpredictab* OR Overt Behavio* OR OBS OR Oppos* OR Parasuicide* OR Attempted Suicide* OR PAS OR RASS OR Resistan* OR Restless* OR RLAS OR Restrain* OR Immobiliz* OR Immobilis* OR Seclu* OR Safety OR Threaten* OR Violen* OR Verbal Preservation OR Motor Preservation OR Emotional Lability) AND ("Amnesia"[mh] OR "Memory Disorders"[mh] OR "Delirium"[mh] OR "Confusion"[mh] OR "Orientation"[mh] OR Amnes* OR Cognitive Retention Disorder* OR "Memory Deficit" OR "Memory Deficits" OR "Memory Disorder" OR "Memory Disorders" OR "Memory Loss" OR "Memory Losses" OR Rancho* OR "Retention Disorder" OR "Retention Disorders" OR RLAS OR Confabulat* OR Dysmnesic OR Disorientat* OR GOAT OR O-Log OR Orientation* OR PTA OR Confus* OR PTCS OR Delirium* OR WPTAS*) NOT MEDLINE[sb]

**H. WHO ICTRP**

Because of the high traffic on this resource for retrieving COVID-19 trials by many users, this service was crashed. We intended to use the following search strategy however this resource was not accessible at the time of search. Since Cochrane Central Register of Controlled Trials (CENTRAL) indexes most of content of this source we searched CENTRAL as temporary solution.

Suggested Search Strategy:

(Trauma OR Traumatic OR Posttrauma OR Posttraumatic OR TBI) AND (Aggression OR Violence OR Agitation OR Self-Injury OR Impulsive OR Compulsive OR Hallucination OR Angry OR Amnesia OR Memory OR Delirium OR Confusion OR Orientation)
