# Supplementary material for: Effectiveness of Non-Pharmacological Interventions for Agitation during Post-Traumatic Amnesia following Traumatic Brain Injury: A Systematic Review
Source: Neuropsychol Rev. 2022 Jun 10;33(2):374–92. doi: 10.1007/s11065-022-09544-5 (PMC10148768; doi:10.1007/s11065-022-09544-5)
Supplement: Supplementary file 3 — Supplementary file3 (DOCX 39 KB) [file 11065_2022_9544_MOESM3_ESM.docx]

**Online Resource 3: Studies Deemed Ineligible at the Full-Text Review Stage**

**Excluded Studies**

| **Study Details** |  | **Reason for Exclusion** |
| --- | --- | --- |
| Alderman, N., Knight, C., & Brooks, J. (2013). Rehabilitation approaches to the management of aggressive behaviour disorders after acquired brain injury. *Brain Impairment*, 14(1), 5-20. |  | Incorrect population; no post-traumatic amnesia |
| Andrewes, H. E., Walker, V., & O'Neill, B. (2014). Exploring the use of positive psychology interventions in brain injury survivors with challenging behaviour. *Brain Injury,* 28(7), 965-971. |  | Incorrect population; no post-traumatic amnesia |
| Bailey, M., Amato, S., & Mouhlas, C. (2009). A creative alternative for providing constant observation on an acute-brain-injury unit. *Rehabilitation Nursing Journal,* 34(1), 11-16. |  | Incorrect population; no post-traumatic amnesia* |
| Clark, A.F., & Davison, K. (1987). Mania following head injury: A report of two cases and a review of the literature. *British Journal of Psychiatry,* 150, 841-844. |  | Incorrect population; no post-traumatic amnesia |
| Eames, P., & Wood, R. (1985). Rehabilitation after severe brain injury: A follow-up study of a behaviour modification approach. *Journal of Neurology, Neurosurgery and Psychiatry*, 48(7), 613-619. |  | Incorrect population; no post-traumatic amnesia |
| Feeney, T. J., & Ylvisaker, M. (1995). Choice and routine: Antecedent behavioral interventions for adolescents with severe traumatic brain injury. *The Journal of Head Trauma Rehabilitation*, 10(3), 67-86. |  | Incorrect population; no post-traumatic amnesia |
| Glassman, L. R. (1991). Music therapy and bibliotherapy in the rehabilitation of traumatic brain injury: A case study. *The Arts in Psychotherapy*, 18(2), 149-156. |  | Incorrect population; no post-traumatic amnesia |
| Gouvier, W. D., Richards, J. S., Blanton, P. D., Janert, K., Rosen, L. A., & Drabman, R. S. (1985). Behavior modification in physical therapy. *Archives of Physical Medicine and Rehabilitation,* 66(2), 113-116. |  | Incorrect population; no post-traumatic amnesia |
| Hegel, M. T. (1988). Application of a token economy with a non-compliant closed head-injured male. *Brain Injury*, 2(4), 333-338. |  | Incorrect population; no post-traumatic amnesia |
| Hitchen, H., Magee, W. L., & Soeterik, S. (2010). Music therapy in the treatment of patients with neuro-behavioural disorders stemming from acquired brain injury. *Nordic Journal of Music Therapy*, 19(1), 63-78. |  | Incorrect population; no post-traumatic amnesia |
| Hufford, B. J., Williams, M. K., Malec, J. F., & Cravotta, D. (2012). Use of behavioural contracting to increase adherence with rehabilitation treatments on an inpatient brain injury unit: A case report. *Brain Injury*, 26(13-14), 1743-1749. |  | Incorrect population; no post-traumatic amnesia |
| Kneale, T. A., & Eames, P. (1991). Pharmacology and flexibility in the rehabilitation of two brain-injured adults. *Brain Injury*, 5(3), 327-330. |  | Incorrect population; no post-traumatic amnesia |
| Kumchy, C. I., & Kores, P. J. (1981). Behavioral management of a neurologically impaired pediatric inpatient. *Archives of Physical Medicine and Rehabilitation*, 62(6), 289-291. |  | Incorrect population; no post-traumatic amnesia* |
| Manchester, D., Hodgkinson A., Pfaff, A., & Nguyen, G. (1997). A non aversive approach to reducing hospital absconding in a head injured adolescent boy. *Brain Injury*, 11(4), 271-278. |  | Incorrect population; no post-traumatic amnesia |
| Manchester, D., Hodgkinson, A., & Casey, T. (1997). Prolonged, severe behavioural disturbance following traumatic brain injury: What can be done. *Brain Injury,* 11(8), 605-618. |  | Incorrect population; no post-traumatic amnesia |
| Matthes, J., & Caples, H. (2013). Ethical issues in using deception to facilitate rehabilitation for a patient with severe traumatic brain injury. *Journal of Head Trauma Rehabilitation*, 28(2), 126-130. |  | Incorrect population; no post-traumatic amnesia |
| Niemeier, J. P., Kreutzer, J. S., Marwitz, J. H., Gary, K. W., & Ketchum, J. M. (2011). Efficacy of a brief acute neurobehavioural intervention following traumatic brain injury: A preliminary investigation. *Brain Injury*, 25(7-8), 680-690. |  | Incorrect population; no post-traumatic amnesia |
| Peters, M. D., Gluck, M., & McCormick, M. (1992). Behaviour rehabilitation of the challenging client in less restrictive settings. *Brain Injury,* 6(4), 299-314. |  | Incorrect population; no post-traumatic amnesia * |
| Rothwell, N. A., LaVigna, G. W., & Willis. T. J. (1999). A non-aversive rehabilitation approach for people with severe behavioural problems resulting from brain injury. *Brain Injury*, 13(7), 521-533. |  | Incorrect population; no post-traumatic amnesia |
| Schleenbaker, R. E., McDowell, S. M., Moore, R. W., Costich, J. F., & Prater, G. (1994). Restraint use in inpatient rehabilitation: Incidence, predictors, and implications. *Archives of Physical Medicine and Rehabilitation*, 75(4), 427-430. |  | Incorrect population; no post-traumatic amnesia* |
| Shapira, M. Y., Chelouche, M., Yanai, R., Kaner, C., & Szold, A. (2001). Tai Chi Chuan practice as a tool for rehabilitation of severe head trauma: 3 case reports. *Archives of Physical Medicine and Rehabilitation*, 82(9), 1283-1285. |  | Incorrect population; no post-traumatic amnesia |
| Slifer, K. J., Cataldo, M. D., Babbitt, R. L., Kane, A. C., Harrison, K. A., & Cataldo, M. F. (1993). Behavior analysis and intervention during hospitalization for brain trauma rehabilitation. *Archives of Physical Medicine and Rehabilitation*, 74(8), 810-817. |  | Incorrect population; no post-traumatic amnesia |
| Stewart, I., & Alderman, N. (2010). Active versus passive management of post-acquired brain injury challenging behaviour: A case study analysis of multiple operant procedures in the treatment of challenging behaviour maintained by negative reinforcement. *Brain Injury*, 24(13-14), 1616-1627. |  | Incorrect population; no post-traumatic amnesia |
| Treadwell, K. J., & Page, T. J. (1996). Functional analysis: Identifying the environmental determinants of severe behavior disorders. *Journal of Head Trauma Rehabilitation*, 11(1), 62-74. |  | Incorrect population; no post-traumatic amnesia |
| Wielenga-Boiten, J. E. & Ribbers, G. M. (2012). Akathisia-rare cause of psychomotor agitation in patients with traumatic brain injury: Case report and review of literature. *Journal of Rehabilitation Research and Development,* 49(9), 1349-1354. |  | Incorrect population; no post-traumatic amnesia |
| Yuen, H. K. (1995). Neurofunctional approach to improve self-care skills in adults with brain damage. *Occupational Therapy in Mental Health*, 12(4), 31-45. |  | Incorrect population; no post-traumatic amnesia |
| Yuen, H. K., & D' Amico, M. (1998). Improved feeding ability in adults with acquired brain damage: Two case studies. *Australian Occupational Therapy Journal,* 45(2), 43-47. |  | Incorrect population; no post-traumatic amnesia |
| Zencius, A. H., & Wesolowski, M. D. (1990). Using stress management to decrease inappropriate behavior in a brain injured adult. *Behavioral Residential Treatment*, 5(1), 61-64. |  | Incorrect population; no post-traumatic amnesia |
| Zencius, A. H., Wesolowski, M. D., Burke, W. H., & McQuade, P. (1989). Antecedent control in the treatment of brain-injured clients. *Brain Injury*, 3(2), 199-205. |  | Incorrect population; no post-traumatic amnesia |
| Zencius, A., Wesolowski, M. D., & Burke, W. H. (1989). Comparing motivational systems with two non-compliant head-injured adolescents. *Brain Injury*, 3(1), 67-71. |  | Incorrect population; no post-traumatic amnesia |
| Alderman, N. (2007). Prevalence, characteristics and causes of aggressive behaviour observed within a neurobehavioural rehabilitation service: Predictors and implications for management. *Brain Injury*, 21(9), 891-911. |  | Incorrect population; no traumatic brain injury |
| Amato, S. P., Salter, J. C., & Mion, L. C. (2006). Physical restraint reduction in the acute rehabilitation setting: A quality improvement study. *Rehabilitation Nursing Journal,* 31(6), 235-241. |  | Incorrect population; no traumatic brain injury |
| Bartolo, M., Bargellesi, S., Castioni, C. A., Intiso, D., Fontana, A., Copetti, M., Scarponi, F., & Bonaiuti, D. (2017). Mobilization in early rehabilitation in intensive care unit patients with severe acquired brain injury: An observational study. *Journal of Rehabilitation Medicine,* 49(9), 715-722. |  | Incorrect population; no traumatic brain injury |
| Beaulieu, C., Wertheimer, J. C., Pickett, L., Spierre, L., Schnorbus, T., Healy, W., Palmer, C., & Jones, A. (2008). Behavior management on an acute brain injury unit: Evaluating the effectiveness of an interdisciplinary training program. *The Journal of Head Trauma Rehabilitation*, 23(5), 304-311. |  | Incorrect population; no traumatic brain injury |
| Galski, T., Palasz, J., Bruno, R. L., & Walker, J. E. (1994). Predicting physical and verbal aggression on a brain trauma unit. *Archives of Physical Medicine and Rehabilitation*, 75(4), 380-383 |  | Incorrect population; no traumatic brain injury |
| Giles, G. M., Scott, K., & Manchester, D. (2013). Staff-reported antecedents to aggression in a post-acute brain injury treatment programme: What are they and what implications do they have for treatment? *Neuropsychological Rehabilitation*, 23(5),732-754. |  | Incorrect population; no traumatic brain injury |
| Imhof, L., Suter-Riederer, S., & Kesselring, J. (2015). Effects of mobility-enhancing nursing intervention in patients with MS and stroke: Randomised controlled trial. *International Scholarly Research Notices*, 1-6. |  | Incorrect population; no traumatic brain injury |
| Janelli, L. M., & Kanski, G. W. (1997). Music intervention with physically restrained patients. *Rehabilitation Nursing*, 22(1), 14-19. |  | Incorrect population; no traumatic brain injury |
| Klages, J., & Dagg, P. (2013). Aggression and brain injury. *Casebook of Neuropsychiatry*, 24-31. |  | Incorrect population; no traumatic brain injury |
| Mohsenian, C., Verhoff, M. A., Rise, M., Heinemann, A., & Puschel, K. (2003). Deaths due to mechanical restraints in institutions for care. *Zeitschrift für Gerontologie und Geriatrie,* 36(4), 266-273. |  | Incorrect population; no traumatic brain injury |
| Mozzoni, M. P. & Hartnedy, S. (2000). Escape and avoidance hypothesis testing using an alternating treatment design. *Behavioral Interventions*, 15(3), 269-277. |  | Incorrect population; no traumatic brain injury |
| Niemeier, J. P., Kreutzer, J. S., & Taylor, L. A. (2005). Acute cognitive and neurobehavioural intervention for individuals with acquired brain injury: Preliminary outcome data. *Neuropsychological Rehabilitation*, 15(2), 129-146. |  | Incorrect population; no traumatic brain injury |
| Schweickert, W. D., Pohlman, M. C., Pohlman, A. S., Nigos, C., Pawlik, A. J., Esbrook, C. L., Spears, L., Miller, M., Franczyk, M., Deprizio, D., Schmidt, G. A., Bowman, A., Barr, R., McCallister, K. E., Hall, J. B., & Kress, J. P. (2009). Early physical and occupational therapy in mechanically ventilated, critically ill patients: A randomised controlled trial. *The Lancet*, 373(9678), 1874-1882. |  | Incorrect population; no traumatic brain injury |
| Slifer, K. J., Cataldo, M. D., & Kurtz, P. F. (1995). Behavioural training during acute brain trauma rehabilitation: An empirical case study. *Brain Injury*, 9(6), 585-593. |  | Incorrect population; no traumatic brain injury |
| Suter-Riederer, S., Mahrer Imhof, R., Kesselring, J., & Imhof, L. (2008). "Care on the ground floor" for agitated people after traumatic brain injury. *Neurologie und Rehabilitation,* 14(2), 70-78. |  | Incorrect population; no traumatic brain injury |
| Vestri, A., Peruch, F., Marchi, S., Frare, M., Guerra, P., Pizzighello, S., Meneghetti, S., Nutbrown, A., & Martinuzzi, A. (2014). Individual and group treatment for patients with acquired brain injury in comprehensive rehabilitation. *Brain Injury*, 28(8), 1102-1108. |  | Incorrect population; no traumatic brain injury |
| Waszynski, C., Veronneau, P., Therrien, K., Brousseau, M., Massa, A., & Levick, S. (2013). Decreasing patient agitation using individualized therapeutic activities. *American Journal of Nursing*, 113(10), 32-41. |  | Incorrect population; no traumatic brain injury |
| Clark-Tippett, D., Palmer, J., & Linden, P. (1987). Case report: Management of dysphagia in a patient with closed head injury. *Dysphagia,* 1(4), 221-226. |  | Incorrect intervention; focus not agitation |
| Corrigan, J. D., Arnett, J. A., Houck, L. J., & Jackson, R. D. (1985). Reality orientation for brain injured patients: Group treatment and monitoring of recovery. *Archives of Physical Medicine and Rehabilitation,* 66(9), 626-630. |  | Incorrect intervention; focus not agitation |
| Dvorkin, A. Y., Zollman, F. S., Beck, K., Larson, E., & Patton, J. L. (2009). A virtual environment-based paradigm for improving attention in TBI. *Rehabilitation Robotics,* 962-965. |  | Incorrect intervention; focus not agitation |
| Formisano, R., Bivona, U., Bruni, F., Vinicola, V., & Villa, M. E. (2003). The family of persons with severe head injury: A valuable resource for education and support. 26(1),117-127. |  | Incorrect intervention; focus not agitation |
| Hartmann, A., Kegelmeyer, D., & Kloos, A. (2018). Use of an errorless learning approach in a person with concomitant traumatic spinal cord injury and brain injury: A case report. *Journal of Neurologic Physical Therapy*, 42(2), 102-109. |  | Incorrect intervention; focus not agitation |
| Hartnedy, S., & Mozzoni, M. P. (2000). Managing environmental stimulation during mealtime: Eating problems in children with traumatic brain injury. *Behavioral Interventions*, 15(3), 261-268. |  | Incorrect intervention; focus not agitation |
| Jia, C. S., Wei, Q. C., He, J., He, C. Q., Li, S. S., Kang, X., Ma, H., & Le, Q. (2013). Effects of early rehabilitation for 51 Lushan victims with traumatic brain injury. *Chinese Journal of Evidence-Based Medicine*, 13(6), 662-665. |  | Incorrect intervention; focus not agitation |
| Larson, E. B., Ramaiya, M., Zollman, F. S., Pacini, S., Hsu, N., Patton, J. L., & Dvorkin, A. Y. (2011). Tolerance of a virtual reality intervention for attention remediation in persons with severe TBI. *Brain Injury*, 25(3), 274-281. |  | Incorrect intervention; focus not agitation |
| Merbitz, C. T., Miller, T. K., & Hansen, N. K. (2003). Cueing and logical problem solving in brain trauma rehabilitation: Frequency patterns in clinician and patient behaviors. *European Journal of Behavior Analysis,* 4(1-2), 45-57. |  | Incorrect intervention; focus not agitation |
| Richardson, B. K. (2020). Promoting functional seated positioning and communication utilising a custom cervical and trunk orthosis in severe traumatic brain injury: A case report. *Disability and Rehabilitation*, 1-7. |  | Incorrect intervention; focus not agitation |
| Spivack, G., Spettell, C. M., Ellis, D. W., & Ross, S. E. (1992). Effects of intensity of treatment and length of stay on rehabilitation outcomes. *Brain Injury*, 6(5), 419-434. |  | Incorrect intervention; focus not agitation |
| Thomas, R., & Mathew, R. (2018). Right median nerve electrical stimulation (RMNS) to improve arousal in acquired brain injury patients a randomised controlled trial. *Neurorehabilitation and Neural Repair*, 32(4‐5), 476. |  | Incorrect intervention; focus not agitation |
| Trevena-Peters, J., McKay, A., Spitz, G., Suda, R., Renison, B., & Ponsford, J. (2018). Efficacy of activities of daily living retraining during posttraumatic amnesia: A randomized controlled trial. *Archives of Physical Medicine and Rehabilitation,* 99(2), 329-337. |  | Incorrect intervention; focus not agitation |
| Trevena-Peters, J., Ponsford, J., & McKay, A. (2018). Agitated behavior and activities of daily living retraining during posttraumatic amnesia. *The Journal of Head Trauma Rehabilitation*, 33(5), 317-325. |  | Incorrect intervention; focus not agitation |
| Williams, K., Christenbury, J., Niemeier, J. P., Newman, M., & Pinto, S. (2020). Is robotic gait training feasible in adults with disorders of consciousness? *The Journal of Head Trauma Rehabilitation,* 35(3), 266-270. |  | Incorrect intervention; focus not agitation |
| Wood, R. L., Winkowski, T., & Miller, J. (1993). Sensory regulation as a method to promote recovery in patients with altered states of consciousness. *Neuropsychological Rehabilitation*, 3(2), 177-190. |  | Incorrect intervention; focus not agitation |
| Arseni, C., Maretsis, M., & Nedelcu, A. (1971). Psychical disorders in recent craniocerebral injuries. *Neurologia, Psihiatria, Neurochirurgia*, 16(2), 109-116. |  | Incorrect outcomes; no relevant measure |
| Duceppe, M., Williamson, D. R., Elliott, A., Para, M., Poirier, M., Delisle, M., Deckelbaum, D., Razek, T., Desjardins, M., Bertrand, J., Bernard, F., Rico, P., Burry, L., Frenette, A. J., & Perreault, M. (2019). Modifiable risk factors for delirium in critically ill trauma patients: A multicenter prospective study. *Journal of Intensive Care Medicine*, 34(4), 330-336. |  | Incorrect outcomes; no relevant measure |
| Edlund, M. J., Goldberg, R. J., & Morris, P. L. P. (1991). The use of physical restraint in patients with cerebral contusion. *The International Journal of Psychiatry in Medicine*, 21(2), 173-182. |  | Incorrect outcomes; no relevant measure |
| Fakhry, S. M., Trask, A. L., Waller, M. A., & Watts, D. D. (2004). Management of brain-injured patients by an evidence-based medicine protocol improves outcomes and decreases hospital charges. *The Journal of Trauma*, 56(3), 492-500. |  | Incorrect outcomes; no relevant measure |
| Irdesel, J., Aydiner, S. B., & Akgoz, S. (2007). Rehabilitation outcome after traumatic brain injury. *Neurocirugia*, 18(1), 5-15. |  | Incorrect outcomes; no relevant measure |
| Kant, R., Coffey, C., Edward, B., & Antonia M. (1999). Safety and efficacy of ECT in patients with head injury. *The Journal of Neuropsychiatry and Clinical Neurosciences*, 11(1), 32-37. |  | Incorrect outcomes; no relevant measure |
| Langhorn, L., Holdgaard, D., Worning, L., Sorensen, J. C., & Pedersen, P. U. (2015). Testing a reality orientation program in patients with traumatic brain injury in a neurointensive care unit. *The Journal of Neuroscience Nursing,* 47(1), 2-10. |  | Incorrect outcomes; no relevant measure |
| Norup, A., Kristensen, K. S., Siert, L., Poulsen, I., & Mortensen, E. L. (2011). Neuropsychological support to relatives of patients with severe traumatic brain injury in the sub-acute phase. *Neuropsychological Rehabilitation*, 21(3), 306-321. |  | Incorrect outcomes; no relevant measure |
| Nott, M. T., Chapparo, C., & Heard, R. (2008). Effective occupational therapy intervention with adults demonstrating agitation during post-traumatic amnesia. *Brain Injury*, 22(9), 669-683. |  | Incorrect outcomes; no relevant measure |
| Stam, D. (2016). Acute treatment of an atypical benign paroxysmal positional vertigo variant post severe traumatic brain injury. *Journal of Acute Care Physical Therapy*, 7(3), 101-112. |  | Incorrect outcomes; no relevant measure |
| Watts, D. D.,Hanfling, D., Waller, M. A., Gilmore, C., Fakhry, S. M., & Trask, A. L. (2004). An evaluation of the use of guidelines in prehospital management of brain injury. *Prehospital Emergency Care*, 8(3), 254-261. |  | Incorrect outcomes; no relevant measure |
| Anonymous (1991). Special report. Electronic article surveillance systems in health care: An update*. Hospital Security and Safety Management*, 11(9), 5-10. |  | Incorrect study design |
| Mintz-Bitman, S., & Gholston, S. M. (1987). Management of the agitated patient in a rehabilitation setting. *Cognitive Rehabilitation*, 5(3), 22-24. |  | Incorrect study design |
| Plylar, P. A. (1989). Management of the agitated and aggressive head injury patient in an acute hospital setting. *The Journal of Neuroscience Nursing*, 21(6), 353-356. |  | Incorrect study design |
| Waxman, R., & Gordon, W. A. (1992). Group-administered cognitive remediation for patients with traumatic brain injury. *NeuroRehabilitation,* 2(3), 46-54. |  | Incorrect study design |
| Werner, G. T. (1994). Early rehabilitation in an intensive-care unit: Stroke and cranio-cerebral trauma as examples. *Physikalische Medizin Rehabilitationsmedizin Kurortmedizin*, 4(6), 229-232. |  | Incorrect study design |
| Williams, J. (2008). Exploring ethically sensitive decision-making in acute hospital care: Using hand-control mittens in adult patients. *Foundation of Nursing Studies Dissemination Series*, 4(8), 1-4. |  | Incorrect study design |
| Wood, L. R., & Alderman, N. (2011). Applications of operant learning theory to the management of challenging behavior after traumatic brain injury. *Journal of Head Trauma Rehabilitation*, 26(3), 202-211. |  | Incorrect study design |
| Bower, J., Catroppa, C., Grocke, D., & Shoemark, H. (2014). Music therapy for early cognitive rehabilitation post-childhood TBI: An intrinsic mixed methods case study. *Developmental Neurorehabilitation*, 17(5), 339-346. |  | Incorrect population; age |
| Hotz, G. A., Castelblanco, A., Lara, I. M., Weiss, A. D., Duncan, R., Kuluz, J. W. (2006). Snoezelen: A controlled multi-sensory stimulation therapy for children recovering from severe brain injury. *Brain Injury*, 20(8), 879-888. |  | Incorrect population; age |
| Pace, G. M., Dunn, E. K., Luiselli, J. K., Cochran, C. R., & Skowron, J. (2005). Antecedent interventions in the management of maladaptive behaviours in a child with brain injury*. Brain Injury*, 19(5), 365-369. |  | Incorrect population; age |
| Swierczynska, A., Klusek, R., Wesolowska, E., & Kacinski, M. (2009). Rehabilitation outcome in children with cranial trauma and hypoxaemia of the brain. *Przeglad Lekarski,* 66(11), 992-995. |  | Incorrect population; age |
| Dvorkin, A. Y., Pacini, S., Hsu, N., & Larson, E. B. (2013). Treatment challenges with profound behaviour disturbance after traumatic brain injury: A case report. *Brain Injury,* 27(7-8), 957-961. |  | Incorrect outcome; no quantitative measure* |
| Saout, V., Gambart, G., Leguay, D., Ferrapie, A. L., Launay, C., & Richard, I. (2011). Aggressive behavior after traumatic brain injury. *Annals of Physical and Rehabilitation Medicine*, 54(4), 259-269. |  | Incorrect outcome; no quantitative measure* |
| Yuen, H. K., & Benzing, P. (1996). Treatment methodology: Guiding of behaviour through redirection in brain injury rehabilitation. *Brain Injury*, 10(3), 229-238. |  | Incorrect outcome; no quantitative measure* |
| Greendyke, R. M., Berkner, J. P., Webster, J. C., & Gulya, A. (1989). Treatment of behavioral problems with pindolol. *Psychosomatics*, 30(2), 161-165. |  | Incorrect intervention; not non-pharmacological |
| Harmsen, M., Geurts, A. C. H., Fasotti, L., & Bevaart, B. J. W. (2004). Positive behavioural disturbances in the rehabilitation phase after severe traumatic brain injury: An historic cohort study. *Brain Injury*, 18(8), 787-796. |  | Incorrect intervention; not non-pharmacological |
| Tang, J. F., Chen, P., Tang, E. J., May, T. A., & Stiver, S. I. (2011). Dexmedetomidine controls agitation and facilitates reliable, serial neurological examinations in a non-intubated patient with traumatic brain injury. *Neurocritical Care*, 15(1), 175-181. |  | Incorrect intervention; not non-pharmacological |

* indicates ‘near-misses’ i.e., studies that met nearly all criteria for inclusion.

**Ongoing Studies**

| **Study Details** |
| --- |
| Investigating the effect of music composition program with family memory remedies on the level of consciousness and cognitive function (2018)  [http://www.who.int/trialsearch/Trial2.aspx?TrialID=IRCT20180602039947N1 2018;():](http://www.who.int/trialsearch/Trial2.aspx?TrialID=IRCT20180602039947N1%202018;():%20) |
| Early rehabilitation of patients with post-traumatic amnesia (2007)  [https://ClinicalTrials.gov/show/NCT00476528 September 2007;():](https://ClinicalTrials.gov/show/NCT00476528%20September%202007;():) |
| Modified environment for agitation in patients with TBI (2015)  [https://ClinicalTrials.gov/show/NCT02524067 September 2015;():](https://ClinicalTrials.gov/show/NCT02524067%20September%202015;():) |
| Multisensory stimulation and enriched environments during post-traumatic amnesia (2015)  [https://ClinicalTrials.gov/show/NCT02792985 August 2015;():](https://ClinicalTrials.gov/show/NCT02792985%20August%202015;():) |
| Effects of multi-sensory stimulation therapy on cognitive impairment in adult patients with traumatic brain injury (2015)  [http://www.who.int/trialsearch/Trial2.aspx?TrialID=TCTR20150128001 2015;():](http://www.who.int/trialsearch/Trial2.aspx?TrialID=TCTR20150128001%202015;():) |

**Conference Abstracts**

| **Study Details** |
| --- |
| Trevena-Peters, J., McKay, A., Spitz, G., Suda, R., & Ponsford, J. (2017). Efficacy of activities of daily living retraining during post-traumatic amnesia. *Brain Injury,* 31(6‐7), 785‐786. |
| Trevena-Peters, J., McKay, A., Spitz, G., Suda, R., Renison, B., & Ponsford, J. (2017). Efficacy of activities of daily living retraining during post-traumatic amnesia: A randomised controlled trial. *Brain Impairment*, 18(3), 334‐335. |
| Trevena-Peters, J., Ponsford, J., & McKay, A. (2018). Agitated behaviour, therapy participation and activities of daily living retraining during posttraumatic amnesia. *Brain Impairment*; 19(3), 312‐313. |
