# Supplementary material for: Effectiveness of Non-Pharmacological Interventions for Agitation during Post-Traumatic Amnesia following Traumatic Brain Injury: A Systematic Review
Source: Neuropsychol Rev. 2022 Jun 10;33(2):374–92. doi: 10.1007/s11065-022-09544-5 (PMC10148768; doi:10.1007/s11065-022-09544-5)
Supplement: Supplementary file 4 — Supplementary file4 (DOCX 19 KB) [file 11065_2022_9544_MOESM4_ESM.docx]

**Online Resource 4: Reference List of Included Studies**

| **Study Details** |
| --- |
| Baker, F. (2001). The effects of live, taped, and no music on people experiencing posttraumatic amnesia. *Journal of Music Therapy*, 38(3), 170-192. |
| Berrol, S. (1988). Risks of restraints in head injury. *Archives of Physical Medicine and Rehabilitation*, 69(7), 537-538. |
| Fluharty, G., & Glassman, N. (2001). Use of antecedent control to improve the outcome of rehabilitation for a client with frontal lobe injury and intolerance for auditory and tactile stimuli. *Brain Injury*, 15(11), 995-1002. |
| Fluharty, G., & Wallat, C. (1997). Modifying the environment to optimize outcome for people with behavior disorders associated with anosognosia. *NeuroRehabilitation*, 9(3), 221-225. |
| Formisano, R., Vinicola, V., Penta, F., Matteis, M., Brunelli, S., & Weckel, J. W. (2001). Active music therapy in the rehabilitation of severe brain injured patients during coma recovery. *Annali Dell'Istituto Superiore Di Sanità,* 37(4), 627-630. |
| Kant, R., Bogyi, A. M., Carosella, N. W., Fishman, E., Kane, V., & Coffey, C. E. (1995). ECT as a therapeutic option in severe brain injury. *Convulsive Therapy*, 11(1), 45-50. |
| Magee, W. L., Baker, F., Daveson, B., Hitchen, H., Kennelly, J., Leung, M., & Tamplin, J. (2011). Music therapy methods with children, adolescents, and adults with severe neurobehavioral disorders due to brain injury*. Music Therapy Perspectives*, 29(1), 5-13. |
| Nielsen, R. M., Olsen, K. S., Lauritsen, A. O. E., & Boesen, H. C. (2014). Electroconvulsive therapy as a treatment for protracted refractory delirium in the intensive care unit: Five cases and a review. *Journal of Critical Care*, 29(5), 881. |
| Park, S., Williams, R. A., & Lee, D. (2016). Effect of preferred music on agitation after traumatic brain injury. *Western Journal of Nursing Research*, 38(4), 394-410. |
| Slifer, K. J., Tucker, C. L., Gerson, A. C., Cataldo, M. D., Sevier, R. C., Suter, A. H., & Kane, A. C. (1996). Operant conditioning for behavior management during posttraumatic amnesia in children and adolescents with brain injury. *The Journal of Head Trauma Rehabilitation*, 11(1), 39-50. |
| Slifer, K. J., Tucker, C. L., Gerson, A. C., Sevier, R. C., Kane, A. C., Amari, A., & Clawson, B. P. (1997). Antecedent management and compliance training improve adolescents' participation in early brain injury rehabilitation. *Brain Injury*, 11(12), 877-889. |
| Wilson, J. L., Hunter, W. M., O'Rourke, J. J. F., & Soble, J. R. (2019). Restructuring blank spaces: The role of cognitive-behavioral interventions for two patients with post-traumatic amnesia after severe traumatic brain injury. *Military Medicine,* 184(1-2), 266-271. |
