# Supplementary material for: Effectiveness of Non-Pharmacological Interventions for Agitation during Post-Traumatic Amnesia following Traumatic Brain Injury: A Systematic Review
Source: Neuropsychol Rev. 2022 Jun 10;33(2):374–92. doi: 10.1007/s11065-022-09544-5 (PMC10148768; doi:10.1007/s11065-022-09544-5)
Supplement: Supplementary file 5 — Supplementary file5 (DOCX 77 KB) [file 11065_2022_9544_MOESM5_ESM.docx]

**Online Resource 5: Critical Appraisal of Included Studies**

Table 1. Critical appraisal results of included randomised controlled trials

| **Study** | **Q1** | **Q2** | **Q3** | **Q4** | **Q5** | **Q6** | **Q7** | **Q8** | **Q9** | **Q10** | **Q11** | **Q12** | **Q13** |
| --- | --- | --- | --- | --- | --- | --- | --- | --- | --- | --- | --- | --- | --- |
| Baker (2001) | Y | N | Y | N | N | N | U | Y | Y | Y | Y | N | N |
| Park et al. (2016) | Y | U | Y | N | N | Y | Y | Y | Y | Y | Y | Y | N |

Y = Yes, N = No, U = Unclear, N/A = Not Applicable.

**Baker (2001)**

| **Risk of bias items** | **Judgement** | **Justification for judgement** |
| --- | --- | --- |
| Q1. Was true randomization used for assignment of participants to treatment groups? | Yes | This study adopted a randomised cross-over design, whereby participants acted as their own control. The order in which they completed each of the three conditions was randomised. Randomisation was computer generated. Assignment of conditions was considered true randomisation and sufficient to meet criteria based on the study design. |
| Q2. Was allocation to treatment groups concealed? | No | The authors indicated that the person conducting randomisation was not separate from the individual allocating participants to study conditions, therefore it is possible that the person allocating the randomised order of the conditions to each participant knew which order of conditions was next in the allocation process. |
| Q3. Were treatment groups similar at baseline? | Yes | There were limited details provided about participant characteristics, however, all participants were early in TBI recovery (i.e., WPTAS score < 9) and were excluded if they had an intellectual disability, psychiatric illness or dementia, which reduces the likelihood that the effect is explained by differences between participants. Furthermore, given the study’s crossover design, participants baseline characteristics are less of a threat to the internal validity of the study, as participants served as their own controls. |
| Q4. Were participants blind to treatment assignment? | No | Blinding to treatment assignment was impossible given the nature of the intervention (environmental), particularly as each participant is exposed to all three conditions. However, given patients were in PTA (i.e., experiencing disorientation and poor memory) it is less likely that the outcomes were impacted by the participants' knowledge of the treatment condition. |
| Q5. Were those delivering treatment blind to treatment assignment? | No | This was an unblinded trial. Therefore, there is a risk that those delivering treatment may have influenced the implementation of the compared treatments and the results of the study may be distorted. |
| Q6. Were outcome assessors blind to treatment assignment? | No | Although it is not clear who conducted the assessments for each outcome, the music therapist who delivered the intervention was unblinded to the treatment groups when rating agitated behaviour pre- and post-intervention. There is therefore a risk that the measurement of this primary outcome may be affected, and the results of the study may be distorted. |
| Q7. Were treatment groups treated identically other than the intervention of interest? | Unclear | The environment was well-controlled and standardised between participants. However, medication usage was not described, and may have influenced agitation levels and other factors, such as level of sedation. Thus, it is plausible that the effect may be explained by other treatments occurring at the same time, such as differences in medication. |
| Q8. Was follow-up complete, and if not, were strategies to address incomplete follow-up utilized? | Yes | Each participant was followed-up following emergence from PTA to determine whether they recalled receiving the music intervention. Given participants were inpatients, attrition is unlikely in this type of study. There is complete knowledge for the entire duration of the trial. |
| Q9. Were participants analysed in the groups to which they were randomized? | Yes | There was no intention-to-treat analysis reported in the present study, however, all participants completed all stages of the study (including follow-up) and thus it is unlikely that this required reporting, particularly given the cross-over design. |
| Q10. Were outcomes measured in the same way for treatment groups? | Yes | Orientation outcomes were measured in the same, consistent manner using the WPTAS and agitation was measured in the same, consistent manner using the ABS. Environmental conditions appeared to be controlled for all patients. Timing of the measurement (pre-intervention for the WPTAS and pre- and post-intervention for the ABS) was consistent across participants. Measurements were timed the same way and followed the same procedures. |
| Q11. Were outcomes measured in a reliable way? | Yes | The WPTAS and ABS have well-established reliability in a TBI population. The WPTAS was administered by the patient's occupational therapist, who was well-versed in its use. The ABS was completed by the music therapist and treating occupational therapist. However, number of raters, training of raters, intra-rater reliability, and inter-rater reliability within the study were not otherwise described. No formal training was described. |
| Q12. Was appropriate statistical analysis used? | No | Statistical power analysis was not reported. The authors did not report whether statistical assumptions were met, despite the ABS tending to be positively skewed. Statistical test selection was seemingly appropriate. The authors conducted many statistical tests with a small sample size (n = 22). Although the authors reported controlling for differences between participants and between days, these control variables were not specified. The authors also created four ABS subscales that may not reflect the underlying factor structure of the ABS. |
| Q13. Was the trial design appropriate, and any deviations from the standard RCT design (individual randomization, parallel groups) accounted for in the conduct and analysis of the trial? | No | The crossover design of this trial is inherently problematic, as PTA is a time-limited state and not a chronic, stable condition. The authors do not report on whether patients emerged from PTA before the entire study protocol was carried out. Further, the authors assertion that "participants were not assigned into experimental and control groups because randomising people with brain injury into cross-matched groups is difficult" could likely have been overcome with a larger sample size. However, the washout period was satisfactory. An RCT where participants are assigned to a single group would be more appropriate. The effects of time were possibly considered in data analysis; however, the authors did not indicate which variables were controlled for in their analyses. |

**Park et al. (2016)**

| **Risk of bias items** | **Judgement** | **Justification for judgement** |
| --- | --- | --- |
| Q1. Was true randomization used for assignment of participants to treatment groups? | Yes | This study adopted a randomised cross-over design, where participants were their own control, and each person was exposed to both treatments. The participants were randomised into two groups (who completed treatment phases in opposite orders) using a computer random number generator, to reduce the risk of carry-over effects from one intervention to the next. |
| Q2. Was allocation to treatment groups concealed? | Unclear | The authors do not address whether concealment of allocation occurred, and it is unclear if the researchers allocating participants to the compared groups were aware of which group was next in the allocation process. |
| Q3. Were treatment groups similar at baseline? | Yes | There were no significant differences in ABS scores across time points between participants in each condition and no significant differences between the two groups according to types of music or significant differences over time. Factors such as age, injury severity and co-existing conditions are less relevant as participants served as their own control. Some patients' baseline agitation levels (prior to intervention) differed noticeably depending on treatment condition, which may reflect the natural recovery process. |
| Q4. Were participants blind to treatment assignment? | No | Participants were not blinded to treatment assignment in this study, given the environmental nature of the intervention. Thus, it is possible that participants behaved differently in response to different interventions. However, participants were in PTA at the time of the intervention and had severe cognitive impairment, thus their awareness of treatment conditions was potentially reduced. |
| Q5. Were those delivering treatment blind to treatment assignment? | No | The researchers setting up the music intervention were not blinded to treatment assignment. There is a risk that they may have behaved differently with participants depending on their treatment condition. |
| Q6. Were outcome assessors blind to treatment assignment? | Yes | Outcome assessors (i.e., raters who scored patients on the ABS) were blinded to the treatment assignments. The researchers did not provide the raters with any information regarding the purpose of the study, to reduce any possible bias in data. Raters would review patient behaviour every hour. It is unlikely that the measurement of outcomes (i.e., ABS scores) was distorted. |
| Q7. Were treatment groups treated identically other than the intervention of interest? | Yes | There was no clear difference between the groups in terms of treatment or care received. It appears that patients received similar care throughout the intervention. The proportion of participants from each group that received the sedative medication is unclear, however, this is presumably alleviated by the absence of data collection on these days. Importantly, each participant acted as their own control, which minimises the effect of differences between treatment groups. |
| Q8. Was follow-up complete, and if not, were strategies to address incomplete follow-up utilized? | Yes | Follow-up was complete; there was complete knowledge for the entire duration of the trial from the moment of random allocation to the end time of the trial for all randomly allocated participants. There was no incomplete follow-up noted and thus no differences regarding loss to follow-up in the compared groups |
| Q9. Were participants analysed in the groups to which they were randomized? | Yes | No intention-to-treat analysis was reported in the present study, however, all participants completed all stages of the study (including follow-up) and thus it is unlikely that this required reporting, particularly given the cross-over design. |
| Q10. Were outcomes measured in the same way for treatment groups? | Yes | It appears that outcomes (i.e., agitation) were measured consistently across the two conditions and over the three time points by a blinded rater. Outcomes were measured at bedside for all patients. Environmental conditions and timing appear to be consistent. Treatment between participants may have varied if patients were provided a non-regular, short-acting sedative, as it would have resulted in data collection being delayed by a day. However, this approach was consistent across participants. |
| Q11. Were outcomes measured in a reliable way? | Yes | The outcomes appeared to be measured in a reliable way. Whilst there was no mention of training of raters, the ABS was scored by the primary researcher and the blinded rater. The trends of data between the two raters were reported as being similar and thus were deemed 'reliable'. No formal inter-rater reliability analysis was conducted and thus there was no statistical substantiation for their claim. |
| Q12. Was appropriate statistical analysis used? | Yes | Statistical power analysis was performed, and appropriate effect sizes were used. The statistical analysis used (mixed-model ANOVA) was appropriate for examining the differences in mean ABS scores according to the order of music selection over time. Independent t-tests were used for carry-over effects and a repeated-measures ANOVA with LSD post hoc analysis was used to determine changes over time and between conditions, as well as the effect of preferred music on the four 'components' of the ABS. The authors did not report whether statistical assumptions were met. Analysis of the subscales of the ABS is questionable, as the authors deviated from the established factor structure of the ABS. The validity of these “components” as representing discrete conceptual factors is in question. |
| Q13. Was the trial design appropriate, and any deviations from the standard RCT design (individual randomization, parallel groups) accounted for in the conduct and analysis of the trial? | No | The crossover RCT design of this trial is inherently problematic, as PTA is a time-limited state, and is therefore not a chronic, stable condition. The 'washout' period of a day was also likely insufficient. An RCT where participants are assigned to a single group would likely be more appropriate. Swapping the order of the conditions and related analyses appeared appropriate and considers the effects of carry-over and time, although it would be helpful to know which participants were assigned to which order of conditions to evaluate possible unreported differences between groups. |

Table 2. Critical appraisal results of eligible quasi-experimental studies

| **Study** | **Q1** | **Q2** | **Q3** | **Q4** | **Q5** | **Q6** | **Q7** | **Q8** | **Q9** |
| --- | --- | --- | --- | --- | --- | --- | --- | --- | --- |
| Formisano et al. (2001) | Y | Y | Y | N | Y | Y | Y | N | N/A |
| Slifer et al. (1996) | Y | Y | Y | N | Y | N | N | Y | Y |
| Slifer et al. (1997) | Y | Y | Y | N | N | N | Y | Y | Y |

Y = Yes, N = No, U = Unclear, N/A = Not Applicable.

**Formisano et al. (2016)**

| **Risk of bias items** | **Judgement** | **Justification for judgement** |
| --- | --- | --- |
| Q1. Is it clear in the study what is the ‘cause’ and what is the ‘effect’ (i.e., there is no confusion about which variable comes first)? | Yes | The cause (music therapy) occurred before the effect (change in agitation levels), thus it is clear which variable was manipulated as a potential cause. |
| Q2. Were the participants included in any comparisons similar? | Yes | Participants were similar in that they were severely injured patients (GCS < 8) during coma recovery. Injury etiology varied. There was also a significant range in coma duration (15-150 days). Most patients who were agitated had sustained a TBI (although only nine patients were initially exhibiting agitation). As each participant served as their own control, the impact of this variability is reduced significantly. |
| Q3. Were the participants included in any comparisons receiving similar treatment / care, other than the exposure or intervention of interest? | Yes | Pharmacological therapy was not changed during the period of music therapy and the intervention was delivered in addition to the routine rehabilitation program. It therefore appears that all patients received care as usual. Furthermore, all patients acted as their own controls, which reduced the impact of any treatment on outcomes. |
| Q4. Was there a control group? | No | There was no separate independent control group; the patients served as their own control. The authors state "the impossibility of a control group depends on the heterogeneity of the clinical condition of comatose patients and of their recovery course that does not lend the individualisation of a comparable control group." Given the nature of TBI recovery, it is likely that agitation may improve when measured at follow-up, and thus a separate independent control group would improve the strength of these findings. |
| Q5. Were there multiple measurements of the outcome both pre and post the intervention / exposure? | Yes | There were five timepoints at which the outcome was measured, including at admission, prior to the rehabilitation program, pre- and post-intervention and at follow-up. However, this data was not provided, which makes it difficult to analyse trends across time points. The authors suggest that positive variations were noted from one month of starting music therapy. |
| Q6. Was follow up complete and if not, were differences between groups in terms of their follow up adequately described and analysed? | Yes | Follow-up was completed and the authors stated that the changes were stable for all patients at follow-up. There was limited detail provided about the outcomes at follow-up. |
| Q7. Were the outcomes of participants included in any comparisons measured in the same way? | Yes | Outcomes were measured in the same way: a music therapist and blind examiner rated video recordings using a semi-quantitative scale of clinical modifications. |
| Q8. Were outcomes measured in a reliable way? | No | No formal measure was used. Outcomes were based on two raters’ judgements as to whether there was improvement, no change, or worsening of psychomotor agitation. Whilst use of two raters improves measurement reliability, the use of a scale that depends on subjective evaluation of outcome measures is not ideal and simple behavioural observation may have been more effective. The authors also did not provide a definition of the behaviours that constitute agitation. |
| Q9. Was appropriate statistical analysis used? | Not applicable | There was no statistical analysis conducted. Authors presented a count of patients who showed improvement in agitation. |

**Slifer et al. (1996)**

| **Risk of bias items** | **Judgement** | **Justification for judgement** |
| --- | --- | --- |
| Q1. Is it clear in the study what is the ‘cause’ and what is the ‘effect’ (i.e., there is no confusion about which variable comes first)? | Yes | The cause (differential reinforcement of appropriate behaviour) occurred before the effect (change in frequency of disruptive behaviours). It is clear which variable was manipulated as a potential cause and that it was manipulated before the occurrence of the effect. Use of a non-concurrent, multiple baseline design (and in one case, an alternating treatment design) was effective in demonstrating the effects of the intervention. |
| Q2. Were the participants included in any comparisons similar? | Yes | All participants had a recent brain injury, at least one behaviour problem that interfered with therapy and were in PTA. Injury severity was relatively similar across participants (GCS ranging from 3-6) and patients’ ages varied slightly (8-16 years). Different brain regions were affected, and days of coma varied considerably. Some patients emerged from PTA during the intervention. Overall, key characteristics that could explain the effect in the absence of the cause were similar among participants. |
| Q3. Were the participants included in any comparisons receiving similar treatment / care, other than the exposure or intervention of interest? | Yes | Treatments were presumably the same for all participants across the baseline and intervention conditions. The use of a baseline condition for each participant reduces the likelihood of the effect being explained by other exposures. |
| Q4. Was there a control group? | No | Whilst there was a baseline condition, there was no separate control group to compare to the treatment group. All patients were exposed to the intervention. |
| Q5. Were there multiple measurements of the outcome both pre and post the intervention / exposure? | Yes | There were multiple measurements of outcome pre- and post-intervention, and the percentage occurrence of target behaviour was captured at baseline and throughout the intervention. PTA assessment using the COAT and GOAT was also conducted at baseline and intervention. |
| Q6. Was follow up complete and if not, were differences between groups in terms of their follow up adequately described and analysed? | No | There was no clear follow-up with patients following the treatment condition, although for some patients who emerged from PTA, behavioural observations and intervention continued following emergence. |
| Q7. Were the outcomes of participants included in any comparisons measured in the same way? | No | There were different problem behaviours measured for different patients (e.g., disruption, elopement) and the behaviours were measured in different ways (e.g., 10-minute time samples, presence and absence of target behaviours across the day). Difference in measurement was due to the number of therapies patients were attending and limited staffing for direct observation. Reliability checks were consistent across patients. |
| Q8. Were outcomes measured in a reliable way? | Yes | The outcomes were measured using direct behavioural observations. The reliability of the behavioural data was assessed by comparing the mean inter-observer percentage agreement between two independent observers (M = 89.8%, range = 78.7-100%). The accuracy of behaviour recordings was checked by a psychology staff observer who intermittently attended therapy sessions and independently documented agreement or disagreement with the therapist’s recordings. There were no recorded disagreements across the 33 sessions that were observed. No formal training was described and raters were not blinded. |
| Q9. Was appropriate statistical analysis used? | Yes | Statistical analysis was basic and included calculation of the mean percentage of target behaviours. |

**Slifer et al. (1997)**

| **Risk of bias items** | **Judgement** | **Justification for judgement** |
| --- | --- | --- |
| Q1. Is it clear in the study what is the ‘cause’ and what is the ‘effect’ (i.e., there is no confusion about which variable comes first)? | Yes | For the minimal demands condition, the cause (compliance training) occurred before the effect (reduced agitation). This is less clear for the usual therapy condition, where patients had emerged from PTA prior to exposure to the condition (PTA emergence may have contributed to improved behaviour). However, given this review’s focus on PTA, cause and effect were clear enough to meet this criterion. Use of a non-concurrent multiple baseline design was effective in demonstrating the effects of intervention for at least one participant. |
| Q2. Were the participants included in any comparisons similar? | Yes | No formal comparison was made between groups, however, participants’ own levels of agitation were compared pre- and post-intervention (although cases 1 and 2 were missing baseline data), which is sufficient to meet this criterion. Participants were also similar in terms of characteristics; the three participants were all female, sustained a severe TBI, were in PTA at the time of the initial intervention, and had a similar RLAS score at the time of intervention. |
| Q3. Were the participants included in any comparisons receiving similar treatment / care, other than the exposure or intervention of interest? | Yes | Patients served as their own control, which reduced the impact of other exposures or treatments affecting the study outcomes. It appears that the same environmental and behavioural strategies were consistently implemented for all participants throughout the duration of the intervention, which increases the likelihood that the intervention can be attributed to the effect. Case 3 was treated with antipsychotic medication for the first 32 days of intervention, although it was noted that this medication did not eliminate agitation or improve therapy compliance. |
| Q4. Was there a control group? | No | There was no control group included in this study. Whilst participants served as their own control (although there was no baseline data for cases 1 and 2), there was no independent control group, which makes it difficult to determine whether the outcomes would have occurred even in the absence of the intervention. |
| Q5. Were there multiple measurements of the outcome both pre and post the intervention / exposure? | No | There were multiple outcome measurements during the two interventions (minimal demands and usual therapy), however, baseline measurements were only taken for case 3. For cases 1 and 2, behavioural psychology consultation was initiated on the first day of scheduled therapies, thus no pre-consultation baseline data was obtained. This makes it difficult to determine any change in agitation as a result of the intervention. |
| Q6. Was follow up complete and if not, were differences between groups in terms of their follow up adequately described and analysed? | No | There was no clear follow-up for participants in this study, including factors such as discharge destination or functional outcomes. Loss to follow-up cannot be calculated given no formal follow-up was conducted. |
| Q7. Were the outcomes of participants included in any comparisons measured in the same way? | Yes | Outcomes were measured in a consistent manner. Whilst the timeline of intervention administration differed among patients (an inherent feature of this study design), the three key outcome measures were recorded consistently for all patients. The ABS was used for all participants, and intervals involving agitated behaviour and therapy attendance were measured in a consistent manner. However, case 3 was the only participant who had a baseline assessment. |
| Q8. Were outcomes measured in a reliable way? | Yes | Outcomes were measured using direct observations including presence of target behaviour over time intervals and scores on the ABS (a validated measurement tool for a TBI population). The ABS was not scored using its original rating scale of severity. Sound justification was provided for the recording of percentage intervals with targeted behaviour and percentage therapy attendance was verified based on therapist progress notes and nursing chart data. There were no reliability estimates but that does not preclude this criterion being met. |
| Q9. Was appropriate statistical analysis used? | Yes | Statistical analysis was basic: use of mean percentage of intervals with targeted disruptive behaviour, mean daily ABS ratings and mean percentage scheduled therapy sessions attended. The analysis was not comprehensive but not considered inappropriate given the study design. |

Table 3. Critical appraisal results of eligible case series

| **Study** | **Q1** | **Q2** | **Q3** | **Q4** | **Q5** | **Q6** | **Q7** | **Q8** | **Q9** | **Q10** |
| --- | --- | --- | --- | --- | --- | --- | --- | --- | --- | --- |
| Fluharty and Wallat (1997) | Y | N | N | N | N | Y | Y | Y | N | N/A |
| Magee et al. (2011) | N | N | N | N | N | Y | N | Y | N | N/A |
| Nielsen (2014) | N | Y | Y | N | N | Y | Y | Y | Y | N/A |
| Wilson (2019) | N | N | N | N | N | Y | Y | N | Y | N/A |

Y = Yes, N = No, U = Unclear, N/A = Not Applicable.

**Fluharty and Wallat (1997)**

| **Risk of bias items** | **Judgement** | **Justification for judgement** |
| --- | --- | --- |
| Q1. Were there clear criteria for inclusion in the case series? | Yes | Inclusion criteria described patients with a global lack of awareness of deficits caused by brain damage (specifically TBI), with confusion and agitation. This criterion is sufficiently clear within the context of the article. |
| Q2. Was the condition measured in a standard, reliable way for all participants included in the case series? | No | Agitation was measured using behavioural observations and qualitative descriptions of agitation; agitated behaviour was not measured in a standard reliable way for both patients. |
| Q3. Were valid methods used for identification of the condition for all participants included in the case series? | No | Patients’ TBI diagnosis was determined based on neuroimaging, whilst anosognosia and agitation were determined qualitatively via observation, rather than using any formal measure. |
| Q4. Did the case series have consecutive inclusion of participants? | No | The authors did not describe their recruitment process, however it is very unlikely to have involved consecutive inclusion of participants, given the cases presented were selected examples for the purposes of demonstrating the effectiveness of specific techniques. |
| Q5. Did the case series have complete inclusion of participants? | No | As per the above criteria, it is unlikely that the case series involved complete inclusion of participants, given the cases presented were selected examples of specific techniques. |
| Q6. Was there clear reporting of the demographics of the participants in the study? | Yes | There was clear reporting of the demographics of the participants in the study in terms of age, sex, injury circumstances and neuroimaging results. There was a lack of detail describing geographic region, education and occupation, however, there is sufficient participant information for this criterion to be met. |
| Q7. Was there clear reporting of clinical information of the participants? | Yes | There was relatively clear reporting of the patient’s head injury and neuroimaging, behavioural outcomes, other relevant conditions (e.g., hemiparesis), and previous admission history. |
| Q8. Were the outcomes or follow up results of cases clearly reported? | Yes | Only qualitative descriptions of agitation were provided, although it appeared that agitated behaviours were counted across the days. The effectiveness of the intervention was determined by descriptions of agitation and subsequent discharge. No adverse events were documented, but a lack of change in anosognosia was acknowledged. Whilst qualitative in nature, the authors describe how the patients progressed over time in terms of agitation and anosognosia, and thus this criterion is met. |
| Q9. Was there clear reporting of the presenting site(s) / clinic(s) demographic information? | No | There was no clear description of the site of the study or study sample, though the study site can be inferred from the author’s affiliation. |
| Q10. Was statistical analysis appropriate? | Not applicable | There was no statistical analysis conducted. It may have been helpful to formally record a count of agitated behaviours. |

**Magee et al. (2011)**

| **Risk of bias items** | **Judgement** | **Justification for judgement** |
| --- | --- | --- |
| Q1. Were there clear criteria for inclusion in the case series? | No | Inclusion criteria was briefly outlined in terms of patients with neurobehavioural disorders following TBI. The case vignettes were divided into adult population, paediatric population and then specifically PTA patients with short-term behavioural disorders. However, no inclusion or exclusion criteria were explicitly provided. |
| Q2. Was the condition measured in a standard, reliable way for all participants included in the case series? | No | The method of measurement of the condition was not described at all in most cases, and not described in detail in the remainder, in terms of TBI diagnosis and agitation. Where agitation and TBI were described, the manner of descriptions differed between participants. |
| Q3. Were valid methods used for identification of the condition for all participants included in the case series? | No | There were no measurement tools used to measure agitation and few used to measure TBI. The validity of identification of agitation is particularly in question due to the subjective nature of this condition. |
| Q4. Did the case series have consecutive inclusion of participants? | No | The authors did not provide comment on whether they included consecutive cases. Given that this study took place over several sites, it is unlikely that consecutive cases were included. |
| Q5. Did the case series have complete inclusion of participants? | No | The authors did not provide comment on whether there was complete inclusion of all participants presenting to rehabilitation. Based on the nature of the examples, it is more likely they were selected by researchers to showcase specific features. |
| Q6. Was there clear reporting of the demographics of the participants in the study? | Yes | Participant demographics that were reported on included age and sex. Education was not described, and geographic region was either the United Kingdom or Australia. There is sufficient information for the purposes of this intervention. |
| Q7. Was there clear reporting of clinical information of the participants? | No | Reporting of TBI was unclear; only two cases mentioned neuroimaging findings or GCS. Severity of TBI was not mentioned in all cases and PTA status was not described for all patients. Cognitive impairments were described where appropriate and agitated behaviours were qualitatively described. No formal measures were used and there were no comorbidities described. |
| Q8. Were the outcomes or follow up results of cases clearly reported? | Yes | For the most part, the authors clearly described the clinical condition post-intervention in terms of the presence of agitation. There was no use of images or figures to aid in explanation. Escalations in agitation (adverse events) were clearly documented and described. No follow-up was undertaken. |
| Q9. Was there clear reporting of the presenting site(s) / clinic(s) demographic information? | No | The authors did not describe the presenting sites in sufficient detail, with the exception of the country where the study was conducted. They did not elaborate on where the intervention was administered or the hospital environment (including whether the service was acute or rehabilitative in nature). |
| Q10. Was statistical analysis appropriate? | Not applicable | No statistical analyses were used, only qualitative analysis. A basic pre-post measure such as the ABS, or behaviour count, would have substantially added to these findings. |

**Nielsen (2014)**

| **Risk of bias items** | **Judgement** | **Justification for judgement** |
| --- | --- | --- |
| Q1. Were there clear criteria for inclusion in the case series? | No | Although inclusion criteria could be discerned from the authors' narrative, they were not explicitly stated as inclusion criteria. No exclusion criteria were noted. It is unclear as to whether the sample characteristics discernible as inclusion criteria reflected all the criteria that were used. |
| Q2. Was the condition measured in a standard, reliable way for all participants included in the case series? | Yes | The study clearly described the method of measurement of agitation and delirium. This is done in a standard and reliable way, using well-validated instruments (i.e., the RASS and CAM-ICU). Diagnosis of TBI is well-substantiated using neuroimaging results, though could have been better captured with greater detail using GCS or LOC. |
| Q3. Were valid methods used for identification of the condition for all participants included in the case series? | Yes | Outcomes were measured using CAM-ICU and RASS (with supplementary qualitative information). Whilst these measures were observed-reported, they are validated instruments, and less likely to impact the validity of outcomes. |
| Q4. Did the case series have consecutive inclusion of participants? | No | The authors did not indicate whether the case series involved consecutive inclusion of participants, but it is unlikely given the specific nature of the examples presented, and the relatively infrequent use of ECT for managing agitation. |
| Q5. Did the case series have complete inclusion of participants? | No | Completeness of this case series is not described, although there is discussion of a sixth case that was managed with ECT but later discontinued the study. As per the above criteria, it is unlikely there was complete inclusion given the specific nature of the examples described. |
| Q6. Was there clear reporting of the demographics of the participants in the study? | Yes | The case series clearly described patient's demographics including age, sex, and brief medical history where relevant. Factors such as geographic region, ethnicity and education were not described but not as pertinent to the measurement of agitation or outcomes of study. |
| Q7. Was there clear reporting of clinical information of the participants? | Yes | There is clear reporting of clinical information of the participants, including injury and mechanism of injury, comorbidities, number of days post-injury, and previous pharmacological and non-pharmacological interventions used (including dosage). |
| Q8. Were the outcomes or follow up results of cases clearly reported? | Yes | Results of the intervention were clearly reported in a table, including number of ECT sessions, resolution of agitation, clinical resolution of delirium, first CAM-ICU negative, as well as status at discharge and follow-up at 30 days and six months. Adverse events were clearly documented. Qualitative information was also provided. However, authors did not report scores for the CAM-ICU or RASS, which would have aided clinical descriptions. |
| Q9. Was there clear reporting of the presenting site(s) / clinic(s) demographic information? | Yes | The authors clearly report the location of the study, although do not describe the environment in detail. There is adequate information provided by the authors to satisfy this criterion. |
| Q10. Was statistical analysis appropriate? | Not applicable | There were no formal statistical analyses conducted. The authors had the opportunity to report and analyse CAM-ICU and RASS data, however, instead relied upon qualitative reporting of treatment efficacy. Quantitative analytical techniques may have been more suitable. |

**Wilson (2019)**

| **Risk of bias items** | **Judgement** | **Justification for judgement** |
| --- | --- | --- |
| Q1. Were there clear criteria for inclusion in the case series? | No | There were no clear criteria for inclusion in the case series (or exclusion criteria). The only criteria discussed was for ‘severe TBI classification.’ No other relevant inclusion criteria were discussed, such as the presence of agitation, PTA, or suitability for CBT. The authors reported an absence of premorbid histories of mood or traumatic disorders, but this was not clearly identified as exclusion criteria. |
| Q2. Was the condition measured in a standard, reliable way for all participants included in the case series? | No | The study described PTA resolution and agitation outcomes, however, agitation outcomes were based on reports from family, staff and psychologists. There is mention of the ABS as a formal measure but the quantitative data were not provided and the tool was not used for all patients. |
| Q3. Were valid methods used for identification of the condition for all participants included in the case series? | No | There was no indication as to whether agitation was identified based on existing definitions or diagnostic criteria. Although the authors described use of the ABS, there was no indication of whether this was used to identify agitation in these participants. Further, all descriptions of agitation were qualitative. Therefore, the researchers did not clearly use a validated instrument to identify agitation and objectivity is potentially compromised. |
| Q4. Did the case series have consecutive inclusion of participants? | No | The authors did not specify whether the two cases included in this case series were consecutive cases. It is likely that these cases were non-consecutive, given the specificity of the intervention and examples provided. |
| Q5. Did the case series have complete inclusion of participants? | No | It is not specified whether all eligible cases were included in this case series, although it is unlikely given the small number of participants included and the specific nature of the intervention. |
| Q6. Was there clear reporting of the demographics of the participants in the study? | Yes | The case series reported relevant demographics, including age, sex, ethnicity (one case only), geographic location and occupation. Education and time post-injury were not reported but were not considered vital to the management of agitation after TBI. |
| Q7. Was there clear reporting of clinical information of the participants? | Yes | There was clear reporting of clinical information about the participants, including the brain injury sustained, the neuropsychological assessment findings and previous admissions. The study also included a clear table of criteria for the classification of brain injury for both cases. |
| Q8. Were the outcomes or follow up results of cases clearly reported? | No | The results of the CBT intervention were clearly reported, indicating a reduction in agitation severity in both cases. However, little detail was provided and no data was described beyond broad statements. Adverse and unanticipated events were not described. |
| Q9. Was there clear reporting of the presenting site(s) / clinic(s) demographic information? | Yes | The specific site at which this research was carried out was described, including an address and an indication that this was a service for military veterans. |
| Q10. Was statistical analysis appropriate? | Not applicable | There were no formal statistical analyses conducted. |

Table 4. Critical appraisal results of eligible case reports

| **Study** | **Q1** | **Q2** | **Q3** | **Q4** | **Q5** | **Q6** | **Q7** | **Q8** |
| --- | --- | --- | --- | --- | --- | --- | --- | --- |
| Fluharty (2001) | Y | Y | Y | Y | Y | Y | Y | N |
| Berrol (1988) | Y | N | N | N | N | Y | Y | Y |
| Kant et al. (1995) | Y | N | Y | Y | Y | Y | Y | Y |

Y = Yes, N = No, U = Unclear, N/A = Not Applicable.

**Fluharty (2001)**

| **Risk of bias items** | **Judgement** | **Justification for judgement** |
| --- | --- | --- |
| Q1. Were patient’s demographic characteristics clearly described? | Yes | The case report described the patient's age, sex, and psychiatric problems. No medications or test results were discussed as there was presumably no significant medical history. The authors also described details of the TBI, including diagnostic results and facilities where the patient was placed. |
| Q2. Was the patient’s history clearly described and presented as a timeline? | Yes | The case report clearly described the history of the patient, including his medical and psychosocial history. No genetic information or family history were presented but this information is not considered crucial to interpreting the study's findings. |
| Q3. Was the current clinical condition of the patient on presentation clearly described? | Yes | The current clinical condition of the patient was described qualitatively in terms of symptoms of PTA and agitation. Frequency and duration of agitation were mostly inferred from qualitative description (no standardised measure). A formal measure of PTA (the GOAT) was used. The authors also described the patient’s medication and neuropsychological assessment results. |
| Q4. Were diagnostic tests or assessment methods and the results clearly described? | Yes | The report provided sufficient information to understand how the patient was assessed. The patient’s TBI diagnosis was substantiated by neuroimaging results and PTA status was substantiated by GOAT scores. However, only one GOAT score is presented, making it difficult to judge the progression of PTA. Although agitation is not quantified, it is well-described qualitatively. No photographs or illustrations were presented. The details provided are considered adequate for assessing treatment efficacy. |
| Q5. Was the intervention(s) or treatment procedure(s) clearly described? | Yes | The authors clearly described behavioural intervention procedures. They only minimally reported on concurrent medication use. More thorough reporting on medication use for behavioural issues would allow the reader to assess whether any changes in regime may have contributed to agitation levels. Qualitative information about interventions conducted was provided, but given how specific the changes were, it would be difficult to replicate or attribute a reduction in agitation to one intervention. Concurrent medication dosages were well-described. |
| Q6. Was the post-intervention clinical condition clearly described? | Yes | The report clearly described the clinical condition post-intervention in terms of the presence or lack of symptoms. The use of a graph to display quantitative results is useful in understanding treatment efficacy. The authors utilised follow up report to their advantage, demonstrating the long-term efficacy of the intervention. |
| Q7. Were adverse events (harms) or unanticipated events identified and described? | Yes | The authors described an unanticipated event and their approach to resolving this issue. No significant adverse events or unanticipated events were described, however, the report highlighted instances where agitation increased. |
| Q8. Does the case report provide takeaway lessons? | No | The case report summarised the background of the condition, and the various impact of the different interventions trialled. It provided some useful information for the use of antecedent control when managing TBI patients with severe deficits in memory, reasoning and insight, as well as hyperesthesia more broadly. However, this report does not appear to present a clear takeaway lesson. Although the authors discuss some aspects of the case (including background literature and the theoretical basis for some findings), they only minimally discuss key lessons learned in terms of clinical practice guidance for clinicians when presented with similar cases. |

**Berrol (1988)**

| **Risk of bias items** | **Judgement** | **Justification for judgement** |
| --- | --- | --- |
| Q1. Were patient’s demographic characteristics clearly described? | Yes | Age, sex, context of injury, diagnosis, treatment and diagnostic test results were well-described. Race, medical history, prognosis and specific medications were not described. The setting was not well described beyond hospital location. The most salient details were provided. |
| Q2. Was the patient’s history clearly described and presented as a timeline? | No | The patient's history was not well described before the time of injury. Medical, family and psychosocial history, including relevant genetic information, were not described. No past interventions for agitated behaviour were described, beyond concurrent sedative medication use. |
| Q3. Was the current clinical condition of the patient on presentation clearly described? | No | The current clinical condition of the patient was described in detail including a thorough overview of symptoms. However, the description of the patient's agitation was minimal and differential diagnoses, uniqueness of the condition, frequency and severity were not described. |
| Q4. Were diagnostic tests or assessment methods and the results clearly described? | No | The assessment and diagnosis of the TBI was adequately described. Assessment of agitation was not described. Assessment of outcomes post-intervention were poorly described. |
| Q5. Was the intervention(s) or treatment procedure(s) clearly described? | No | The nature of non-pharmacological intervention was well described, however, time post-injury, frequency and duration with which it was applied were not fully described. The concurrent use of sedating medication was not described in detail. The intervention was described in terms of types of physical restraint used and when they were used. |
| Q6. Was the post-intervention clinical condition clearly described? | Yes | Clinical condition post-intervention was clearly described in terms of medical status, medical intervention and follow-up. The changes in clinical condition post-intervention because of harms was well described. |
| Q7. Were adverse events (harms) or unanticipated events identified and described? | Yes | Harms to the patient were well-documented and described and followed-up for an extended period. |
| Q8. Does the case report provide takeaway lessons? | Yes | The report summarises key lessons (i.e., assessing safety issues and potential alternative measures to physical restraints). The report also highlighted that a confusional state alone does not justify the use of restraints. The author summarises key lessons learned from the case and offers clinical practice guidance for clinicians when presented with similar cases. |

**Kant et al. (1995)**

| **Risk of bias items** | **Judgement** | **Justification for judgement** |
| --- | --- | --- |
| Q1. Were patient’s demographic characteristics clearly described? | Yes | The case report described the patient’s age, sex and handedness and highlighted no prior history of any neurologic or psychiatric illness. The assessment and treatment of the head injury were also described. Previous behavioural and medical interventions were described. |
| Q2. Was the patient’s history clearly described and presented as a timeline? | No | No previous patient history was described, apart from the absence of any neurological or psychiatric illness. It would be helpful to provide details regarding the patient’s occupation, education and family history. |
| Q3. Was the current clinical condition of the patient on presentation clearly described? | Yes | The patient's head injury is well described. Agitation is described in detail, including at admission, and following behavioural intervention, restraint and medical interventions. The case report also described the patients’ cognition, speech, orientation status, etc. The frequency and severity of agitation were described. Differential diagnoses were discussed. The authors did not comment on the uniqueness of the case presentation. |
| Q4. Were diagnostic tests or assessment methods and the results clearly described? | Yes | All appropriate tests were conducted to confirm a diagnosis of TBI, and the authors included an image of the patient's neuroimaging results. The case report also provided a clear description of various diagnostic tests used to establish an underlying cause for delirium. The authors did not objectively measure the patient's levels of agitation but provided an adequate qualitative description of this behaviour. |
| Q5. Was the intervention(s) or treatment procedure(s) clearly described? | Yes | The authors clearly described treatment procedures, enabling clear understanding of the treatment protocol. This included description of previous unsuccessful interventions (behavioural and pharmacological) and the ECT. ECT course, frequency and duration were described, and details of the ECT procedure were provided. Anaesthesia management was described in detail and seizure threshold values described and referenced. The report also described medication used throughout ECT treatment, including drug type, dosage, frequency and purpose. Side effects of ECT were described, namely mild confusion and worsening language deficits, as well as for pharmacotherapy. |
| Q6. Was the post-intervention clinical condition clearly described? | Yes | Agitation symptoms were described qualitatively and extended to cognitive abilities more broadly. Also, the GOAT, MMSE and NRS were conducted at admission, pre- and post-ECT and at follow-up, providing detailed information about the patient's pre- and post-clinical condition. Tables were clear and helpful in providing readers with relevant information. A graph may have been useful to visualise improvement. |
| Q7. Were adverse events (harms) or unanticipated events identified and described? | Yes | Adverse events including mild confusion and worsening language deficits were described following the ECT course, but these were noted to have subsided at the end of the treatment course. |
| Q8. Does the case report provide takeaway lessons? | Yes | The authors briefly summarised the key lesson learned from the case. They summarised important factors for clinicians to consider when undertaking a similar procedure such as looking out for underlying causes of delirium and use of non-pharmacological and pharmacological avenues to treat agitation prior to ECT. |
